# Hydrophobic Drug Delivery Platforms Based on Covalent Organic Frameworks for Combined Treatment of Alzheimer’s Disease

**DOI:** 10.3390/ijms262110803

**Published:** 2025-11-06

**Authors:** Yun Zhao, Ziwei Wang, Enpeng Xi, Fuming Yang, Nan Gao

**Affiliations:** Key Laboratory of Polyoxometalate and Reticular Material Chemistry of Ministry of Education, Faculty of Chemistry, Northeast Normal University, Changchun 130024, China; zhaoyun@nenu.edu.cn (Y.Z.); wangzw298@nenu.edu.cn (Z.W.);

**Keywords:** Alzheimer’s disease, neuroinflammation, drug carrier, curcumin, benzofuroxan

## Abstract

Alzheimer’s disease (AD) is a complex neurodegenerative disease. The pathogenesis of AD remains incompletely understood. It is characterized by a variety of neuropathological changes, including neuroinflammation, neuronal loss and synaptic damage. Multiple pathological changes make achieving good therapeutic effects with a single drug treatment difficult, and using multiple drugs for combination therapy is currently the most effective method. Currently, the mainstay drugs used for AD treatment are hydrophobic drugs, such as curcumin, donepezil, and resveratrol. Because hydrophobic drugs cannot dissolve in bodily fluids and often aggregate or precipitate, their efficacy is greatly reduced. Therefore, there is an urgent need for a drug carrier that can effectively load and continuously release drugs. However, currently, there are few drug carriers that can achieve efficient co-loading of multiple hydrophobic drugs. Therefore, three of two-dimensional imine covalent organic frameworks (COFs) with different monomers were synthesized through rational design and screening. These three synthesized COFs are simultaneously loaded with curcumin (CUR) and benzofurazan (BZ) to achieve combined therapy. The results indicate that among this series of synthesized COFs, the COF synthesized from 4,4′,4″-(1,3,5-Triazine-2,4,6-triyl) trianiline and benzene-1,3,5-tricarboxaldehyde (COF-TB) exhibits optimal hydrophobic drug-loading capacity, enabling effective co-loading of CUR and BZ (BC@COF-TB). After treatment with BC@COF-TB, the cognitive function of 5×FAD mice was significantly improved. The COF platform provides a new way to deliver hydrophobic drugs for AD treatment.

## 1. Introduction

Alzheimer’s disease (AD) is a neurodegenerative disease. According to statistics, the global prevalence of AD is as high as 24 million, and the number of patients is expected to reach more than 139 million by 2050. Total annual health care costs for patients with dementia, including long-term care and end-of-life care, are projected to nearly quadruple from USD 305 billion in 2020 to more than USD 1.1 trillion by 2050, placing a heavy burden on families and society [1,2,3,4,5]. Although various hypotheses about the pathogenesis of AD have been proposed, such as the amyloid cascade hypothesis, tau protein hypothesis, inflammation hypothesis, and cholinergic injury theory, the pathogenesis of AD has not been fully elucidated, which limits the therapeutic effect of clinical trial drugs [6]. The reason is that AD can cause various neuropathological injuries, and the reversal and recovery from the neuropathological changes accumulated during the very long course of AD through treatment with a single drug are difficult. Therefore, designing a multi-drug therapeutic system may be an effective way to treat AD [7,8,9,10].

Currently, the mainstay drugs used for AD treatment are hydrophobic drugs, such as curcumin, donepezil, and resveratrol [11]. Hydrophobic drugs contain a large number of hydrophobic groups, making them difficult to dissolve in body fluids and be transported to the affected area. They often aggregate or precipitate, resulting in a significant reduction in their efficacy. Traditional methods use organic solvents or surfactants to forcibly dissolve drugs, but they can lead to significant toxic side effects. Meanwhile, current drug carriers mainly include liposomes, micelles, carbon nanotubes, quantum dots, gold nanoparticles, and dendritic macromolecules. Due to their low drug loading capacity for hydrophobic drugs, poor biocompatibility, and rapid release rates, these traditional drug carriers face limitations in their application for in vivo therapy [12,13,14,15,16,17].

Even worse, currently, there are few drug carriers that can achieve efficient co-loading of multiple hydrophobic drugs. Therefore, the development of an effective hydrophobic multi-drug carrier for the treatment of AD is urgently needed [18,19]. Covalent organic frameworks (COFs), which are porous materials, can serve as effective drug carriers due to their large specific surface area and good biocompatibility. Currently, COFs have received considerable attention as drug delivery carriers. For example, the acid-responsive COF hydrogel synthesized by Meng et al. can be used to load the hydrophobic drug DOX for treating lung cancer [20]. Asadi et al. introduced various functional groups into the synthesized COF to increase hydrophilicity, thereby improving the delivery ability of DOX [21]. Vyas et al. synthesized a COF based on imine bonds to load quercetin for cancer treatment [22]. Ge et al. designed a photoactivated COF loaded with chlorin e6 and tirapamine for in vivo drug delivery for cancer treatment [23]. Zhang et al. used polyethylene glycol-modified COF for the in vivo delivery of DOX as a targeted therapy for cancer [24]. COFs exhibit excellent performance in drug delivery, making them a promising approach as ideal hydrophobic drug carriers for treating AD [25,26].

Therefore, in this study, three two-dimensional imine COFs (COF-TB, COF-TP, and COF-TC) with different monomers were synthesized through rational design and screening, which could load curcumin (CUR) and benzofuroxan (BZ) simultaneously, and had the potential to be developed into multi-target combined therapy carriers. The screening results showed that BC@COF-TB could achieve 9.04% and 10.78% loading of CUR and BZ and had the best drug loading and release ability. In animal experiments, COF-TB loaded with the two hydrophobic drugs effectively alleviated the cognitive impairment of 5×FAD mice. This proves once again that COF-TB is an effective hydrophobic drug carrier and has great potential in the treatment of AD.

## 2. Results

### 2.1. Synthesis and Characterization of COFs

**Figure 1:**
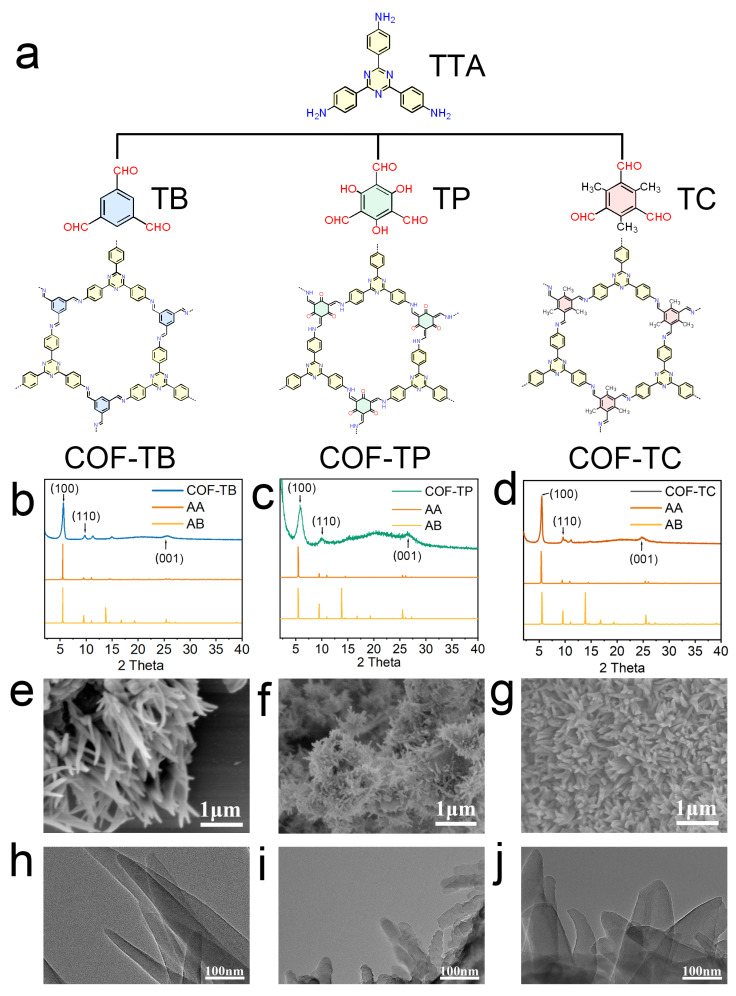
Synthesis and characterization of COFs. (**a**) Structures of COF-TB, COF-TP, and COF-TC and their corresponding monomers. (**b**–**d**) PXRD patterns of COF-TB, COF-TP, and COF-TC. (**e**–**g**) SEM images of COF-TB, COF-TP, and COF-TC. (**h**–**j**) TEM images of COF-TB, COF-TP, and COF-TC.

In this study, COF-TB, COF-TP, and COF-TC were synthesized using the solvothermal method. All three COFs were synthesized through a Schiff base reaction. The structural diagrams of these three COFs are shown in Figure 1. The product was a brown or yellow powder (Figure S1). Then, we performed a series of characterizations of the three COFs we synthesized. The powder X-ray diffraction (PXRD) results are shown in Figure 1b–d. The diffraction peaks of COF-TB at 5.62°, 9.74°, and 25.62° correspond to the (100), (110), and (001) crystal planes [27,28]. The diffraction peaks of COF-TP at 5.82°, 10.0°, and 26.46° represent the (100), (110), and (001) crystal planes [29,30]. The diffraction peaks of COF-TC at 5.50°, 9.62°, and 24.98° represent the (100), (110), and (001) crystal planes [31,32]. The crystal structures and stacking modes of these COFs (Figure S2) were predicted using the Material Studio program. The crystal structures of COF-TB, COF-TP, and COF-TC are detailed in Tables S1–S3. The comparison between the simulation results and the experimental data shows that these three COFs are AA-stacked hexagonal structures [33,34,35]. After treatment with water, ethanol, HCl (pH = 5), NaOH (pH = 10), and PBS for one week, the crystal structures of the COFs remained intact (Figure S3), indicating their good stability in solvents. Fourier transform infrared spectroscopy (FT-IR, Figure S4) shows that COF-TB, COF-TP and COF-TC all have a characteristic stretching vibration band for the C = N bond at 1584 cm^−1^. Based on all the characterization results mentioned above, it is proven that the three COFs have been successfully synthesized [36,37,38,39,40,41].

Isothermal nitrogen adsorption and desorption experiments were performed on these three COFs at 77 K to analyze their porosity [42,43,44,45,46]. As shown in Figure S5, the BET specific surface areas of COF-TB, COF-TP, and COF-TC are 1195.548, 816.701, and 1004.483 m^2^·g^−1^, respectively. The pore sizes calculated based on BET are 0.966, 1.169, and 1.007 nm, respectively. These results are similar to previous research findings [27,28,29,30,31,32]. They indicate that the skeletons of these three COFs have large specific surface areas and a uniform porosity matching the corresponding crystal structure. Then, the surface morphology and microstructure of COFs were studied using scanning electron microscopy (SEM) and transmission electron microscopy (TEM). The images (Figure 1e–j) show that COF-TB has an irregular needle-like structure, and COF-TP and COF-TC show a uniform nanoflower morphology.

### 2.2. Drug Loading Capacity of COFs

The in vivo application of some excellent anti-inflammatory drugs, such as CUR and BZ, is limited due to their insolubility. CUR is a natural component of turmeric, which is a polyphenol. It has anti-inflammatory and antioxidant biological activities. It is an excellent reactive oxygen species scavenger that can effectively remove superoxide and hydrogen peroxide. However, CUR has low solubility (<1 μg·mL^−1^) and a low utilization rate in the body, which limits its application [47,48,49,50]. BZ is an important organic nitrogen heterocyclic compound that can produce nitric oxide in the body through the action of cysteine and other thiol compounds [51,52,53,54]. The produced nitric oxide can be used to control neuroinflammation in the brain [55,56,57,58,59,60]. Unfortunately, the low solubility of BZ limits its application in vivo. Therefore, active development of carriers that can load poorly soluble drugs can solve the dilemma of hydrophobic drug applications in vivo, effectively broaden the application scenarios of these poorly soluble drugs, and provide new ideas for treating various inflammatory diseases.

**Figure 2:**
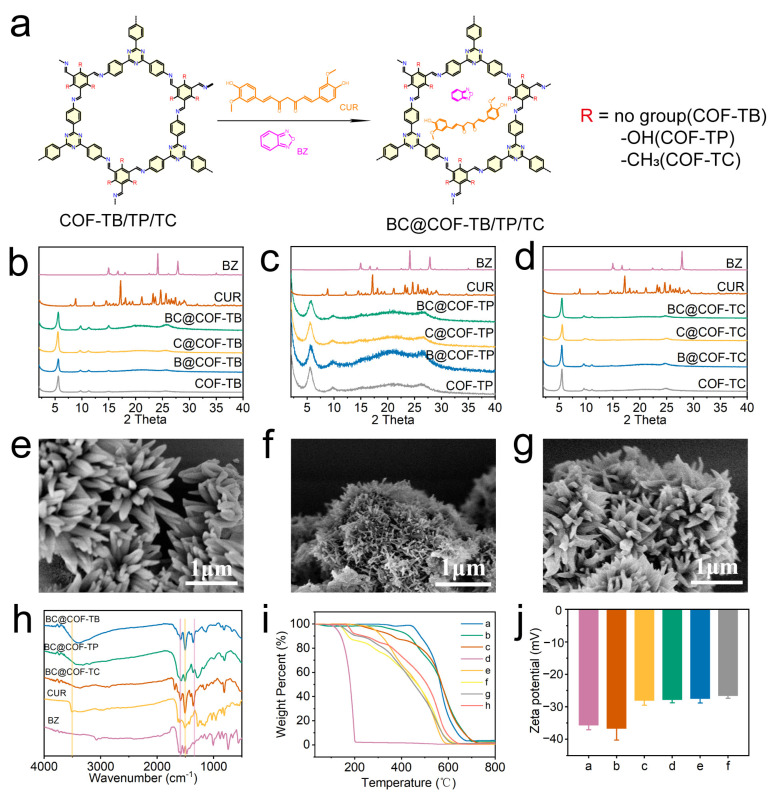
Synthesis and characterization of drug-loaded COFs. (**a**) Diagram of the drug loading process. (**b**–**d**) PXRD patterns of COF-TB, COF-TP, and COF-TC after drug loading. (**e**–**g**) SEM images of BC@COF-TB, BC@COF-TP, and BC@COF-TC. (**h**) FT-IR patterns of BC@COF-TB, BC@COF-TP, and BC@COF-TC (the purple line represents the characteristic stretching vibration band of BZ, and the yellow line represents the characteristic stretching vibration band of CUR). (**i**) TGA results (a, COF-TB; b, COF-TP; c, COF-TC; d, BZ; e, CUR; f, BC@COF-TB; g, BC@COF-TP; h, BC@COF-TC). (**j**) Zeta potential diagrams (a, COF-TB; b, COF-TP; c, COF-TC; d, BC@COF-TB; e, BC@COF-TP; f, BC@COF-TC). (*n* = 3).

The drug loading capacities of CUR and BZ in COF-TB, COF-TP, and COF-TC were comprehensively analyzed and measured separately. Afterwards, the ability of three COFs to jointly load CUR and BZ was further analyzed (Figure 2a).

Firstly, the three COFs loaded with hydrophobic drugs were characterized to ensure that the structure of the COFs did not change significantly after drug loading. Figure 2b–d show the PXRD patterns of three types of COFs loaded with BZ and CUR. The results showed that the XRD peaks of the three COFs remained consistent before and after drug loading, indicating that there were no significant changes in the structures of the three COFs after drug loading. The SEM images (Figure 2e–g and Figure S6) show that the morphology of COF-TB, COF-TP, and COF-TC remained unchanged before and after drug loading, indicating that the structure of COF was not damaged during drug loading.

The three COFs loaded with drugs were analyzed using FT-IR, and the results are shown in Figure 2h and Figure S7. The stretching vibration bands of O-H and C = O in CUR at 3510 cm^−1^ and 1506 cm^−1^, and the stretching vibration bands of Furazan ring in BZ at 1330 cm^−1^ and 1600 cm^−1^ are characteristic bands of the two drugs. After the three COFs were loaded with CUR or BZ, the corresponding stretching vibration bands of the loaded drugs appeared. When three COFs were co-loaded with the two drugs, characteristic stretching vibration bands of both drugs appeared simultaneously. The FT-IR results showed that the three COFs achieved separate loading and co-loading of the two drugs, respectively.

The drug loading capacity and thermal stability of the COFs were investigated through a thermogravimetric analysis (TGA). As shown in Figure 2i and Figure S8, COF-TB, COF-TP, and COF-TC still retain over 80% of their weight at 500 °C, indicating that these three COFs have good thermal stability. The TGA results for COF-TB, COF-TP, and COF-TC loaded with BZ and CUR show three processes of mass loss. The weight loss occurring at 150–200 °C is caused by the decomposition of BZ. The weight loss occurring between 250 and 360 °C is caused by the decomposition of CUR. The weight loss at 500–800 °C is the result of COF decomposition. Meanwhile, the zeta potentials of COF-TB, COF-TP, and COF-TC were measured, which were −35.7, −36.7, and −28.1 MV, respectively. When three COFs were loaded with BZ or CUR, the potential increased slightly. The same phenomenon was observed when three COFs were loaded with both BZ and CUR (Figure 2j and Figure S9). The TGA and zeta potential results once again demonstrate that these three COFs can load CUR and BZ. The above characterization results showed that we successfully prepared the hydrophobic drug delivery systems based on COF-TB, COF-TP, and COF-TC.

### 2.3. Loading and Release of BZ and CUR from COFs

**Figure 3:**
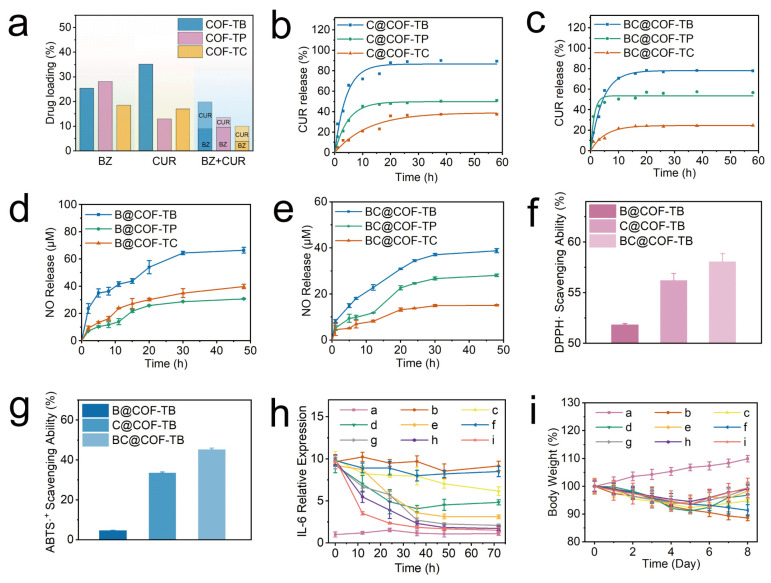
(**a**) Drug loading in different systems. (**b**,**c**) CUR release curves for the different materials. (**d**,**e**) Nitric oxide release curves for the different materials. (**f**) DPPH· radical scavenging ability. (**g**) ABTS radical scavenging ability. (**h**) Relative IL-6 expression. (**i**) Weight changes in the mice from each group. (a, control; b, LPS; c, LPS + BZ; d, LPS + CUR; e, LPS + BZ + CUR; f, LPS + COF-TB; g, LPS + B@COF-TB; h, LPS + C@COF-TB; i, LPS + BC@COF-TB groups) (*n* = 5).

The loading capabilities of CUR and BZ in the COFs were then fully explored through loading and release experiments. As shown in Figure 3a and Table S4, the loading capacities of BZ and CUR in the three COFs were determined. In the single-loaded BZ system, BZ loading in COF-TB, COF-TP, and COF-TC was 25.40%, 28.15%, and 18.56%, respectively. In the single CUR-loaded system, CUR loading in COF-TB, COF-TP, and COF-TC was 35.17%, 12.96%, and 17.00%, respectively. Finally, in the BZ and CUR dual drug delivery system, BZ and CUR loading in COF-TB, COF-TP, and COF-TC were 9.04%, 9.66%, and 3.85%, and 10.78%, 3.88%, and 6.17%, respectively. The above results indicate that COF-TB has the strongest loading capacity for CUR, which may be due to the introduction of other functional groups in COF-TP and COF-TC that cause steric hindrance within the pore size, limiting the effective loading space within the pore. The loading capacity of BZ in COF-TP is slightly higher than that in COF-TB, which may be due to the easy hydrogen bonding interaction between BZ and the hydroxyl groups carried by COF-TC, thereby improving the loading capacity (Figure S10). COF-TC has the worst load capacity for both CUR and BZ. The results indicate significant differences in loading capacity caused by the different functional groups carried by COFs. When the three COFs are co-loaded with the two drugs, the drug loading capacity of the three COFs decreases. The reason may be due to the competition between BZ and CUR within the pores, leading to a decrease in the drug loading capacity. Overall, COF-TB has the best loading capacity when CUR and BZ are loaded together.

The release of CUR was monitored using an ultraviolet–visible spectrophotometer. It can be seen from Figure 3b that in the first five hours, the amounts of CUR released from C@COF-TB, C@COF-TP, and C@COF-TC were 65.86%, 31.29%, and 12.18%, respectively. C@COF-TB and C@COF-TP stopped releasing CUR at about 10 h, whereas C@COF-TC stopped releasing CUR at about 20 h. The cumulative amounts of CUR released from C@COF-TB, C@COF-TP, and C@COF-TC were 89.39%, 51.13%, and 37.43%, respectively. Similarly, in Figure 3c, the release of CUR from BC@COF-TB, BC@COF-TP, and BC@COF-TC in the first five hours was 58.75%, 47.14%, and 12.22%, respectively. BC@COF-TB and BC@COF-TC stopped releasing CUR at about 15 h, and BC@COF-TP stopped releasing CUR at about 5 h. The cumulative release rates of BC@COF-TB, BC@COF-TP and BC@COF-TC were 77.88%, 56.67%, and 24.70%, respectively. It can be seen that in the single-loaded CUR and double-loaded drug systems, COF-TB exhibits the highest CUR release when used as a drug carrier.

BZ is a thiol-dependent nitric oxide donor. To study nitric oxide release, the curve of nitric oxide release was established using the Griess method in PBS with cysteine. As shown in Figure S11, taking BC@COF-TB as an example, BC@COF-TB alone does not produce obvious nitric oxide in PBS, but can produce a large amount of nitric oxide in cysteine-containing PBS or Dulbecco’s Modified Eagle Medium (DMEM), which can achieve intelligent responsive release of nitric oxide. It can be seen in Figure 3d that the cumulative nitric oxide release from B@COF-TB, B@COF-TP, and B@COF-TC within 48 h was 66.4, 30.8, and 39.8 μM, respectively. Although the loading capacity of BZ in COF-TP is slightly higher than that in COF-TB, the amount released from COF-TP is much lower than that from COF-TB. This once again confirms the interaction between COF-TP and BZ, which increases the loading capacity but seriously affects its release ability. The cumulative amounts of nitric oxide released from BC@COF-TB, BC@COF-TP, and BC@COF-TC within 48 h were 38.8, 28.1, and 15.1 μM, respectively (Figure 3e). In summary, considering the loading capacity of CUR and BZ, the release of CUR, and the generation of nitric oxide, COF-TB has the optimal loading capacity for CUR and BZ. Therefore, COF-TB was chosen as the drug delivery platform for further experimental research.

### 2.4. Antioxidant Properties of B@COF-TB, C@COF-TB, and BC@COF-TB

BZ and CUR have antioxidant and anti-inflammatory properties. Therefore, we investigated the antioxidant activities of COF-TB, COF-TB, and @ COF-TB through a DPPH radical scavenging assay and ABTS radical scavenging assay. The purple 2,2-diphenyl-1-picrylhydrazyl radical (DPPH·) reacted with the antioxidant to become colorless DPPH·, and the UV absorbance decreased. According to this characteristic, we measured the DPPH· free radical scavenging rates of B@COF-TB, C@COF-TB, and BC@COF-TB using a UV-visible spectrophotometer, which were 51.84%, 56.23%, and 58.08%, respectively (Figure 3f and Figure S12). BC@COF-TB had the highest DPPH· free radical scavenging rate, indicating that COF-TB had the highest antioxidant activity when co-loaded with BZ and CUR, which was mainly attributed to the effect of CUR. The ABTS radical scavenging rates of B@COF-TB, C@COF-TB, and BC@COF-TB were 4.57%, 33.70%, and 45.37%, respectively, among which BC@COF-TB also had the highest rate (Figure 3g and Figure S13). The above results indicate that BC@COF-TB has the best antioxidant capacity.

Overall, COF-TB has the highest loading capacity for hydrophobic drugs, which may be due to the introduction of other functional groups in COF-TC and COF-TP, resulting in steric hindrance and limited effective loading space within the pore. The loading capacity of COF-TP on BZ is slightly higher than that of COF-TB, which may be due to the easy hydrogen bonding interaction between BZ and the hydroxyl groups on COF-TC, thereby improving the loading capacity. However, the presence of hydrogen bonds hinders the release of BZ. COF-TC has the lowest loading capacity for both CUR and BZ. The results indicate significant differences in loading capacity caused by different functional groups carried by COFs. When three COFs are co-loaded with the two drugs, the drug loading capacity of the three COFs decreases significantly. The reason may be due to the competition between BZ and CUR within the pores, leading to a decrease in the drug loading capacity. Overall, when CUR and BZ are loaded together, COF-TB has the highest loading capacity.

### 2.5. In Vivo Properties of B@COF-TB, C@COF-TB and BC@COF-TB

The most basic requirement for in vivo experiments is to ensure the safety of the materials. Before the in vivo experiments, we evaluated the biosafety of the materials. Firstly, the blood compatibility of the materials was evaluated. The hemolysis test results showed that different concentrations of COF-TB and BC@COF-TB had a hemolytic effect of less than 5%, which meets the safety requirements (Figure S14). After the intraperitoneal injection of COF-TB or BC@COF-TB into mice, there was no significant change in their body weight (Figure S15). Subsequently, the main organs and blood of the mice were collected for analysis. No significant organ damage was observed in the H&E-stained sections of the main organs (Figure S16). The results of blood routine and blood biochemical analyses of mice showed that all indicators were within the normal range (Figure S17). These results indicate that the COF carriers have good biocompatibility.

#### 2.5.1. Anti-Inflammatory Effects of B@COF-TB, C@COF-TB, and BC@COF-TB

To study the anti-inflammatory effects of B@COF-TB, C@COF-TB, and BC@COF-TB, the lipopolysaccharide (LPS)-induced inflammation model was used. After equilibrating the purchased male ICR mice in a barrier environment for one week, the mice were randomly divided into nine groups, each containing five mice. The mice were injected with LPS for a total of five days to establish an inflammation model, and the mice were treated for three days according to the groups. After treatment, the mice were euthanized and blood samples were collected for analysis. Figure 3h and Figure S18 show that the serum interleukin-6 (IL-6) content of mice in the BC@COF-TB group was significantly lower than that in the LPS group, and the anti-inflammatory effect of the free drug and single drug-loaded COF was lower than that of double drug-loaded COF, indicating that the double drug-loaded COF had a good anti-inflammatory effect. The body weight changes in mice were continuously detected during the establishment of the inflammation model and treatment (Figure 3i). The weight gain of the BC@COF-TB group of mice was the highest after treatment, which once again proved that COF of dual-loaded hydrophobic drugs delivers superior anti-inflammatory effects compared to being loaded with a single drug.

#### 2.5.2. Therapeutic Effect of BC@COF-TB on AD Mice

**Figure 4:**
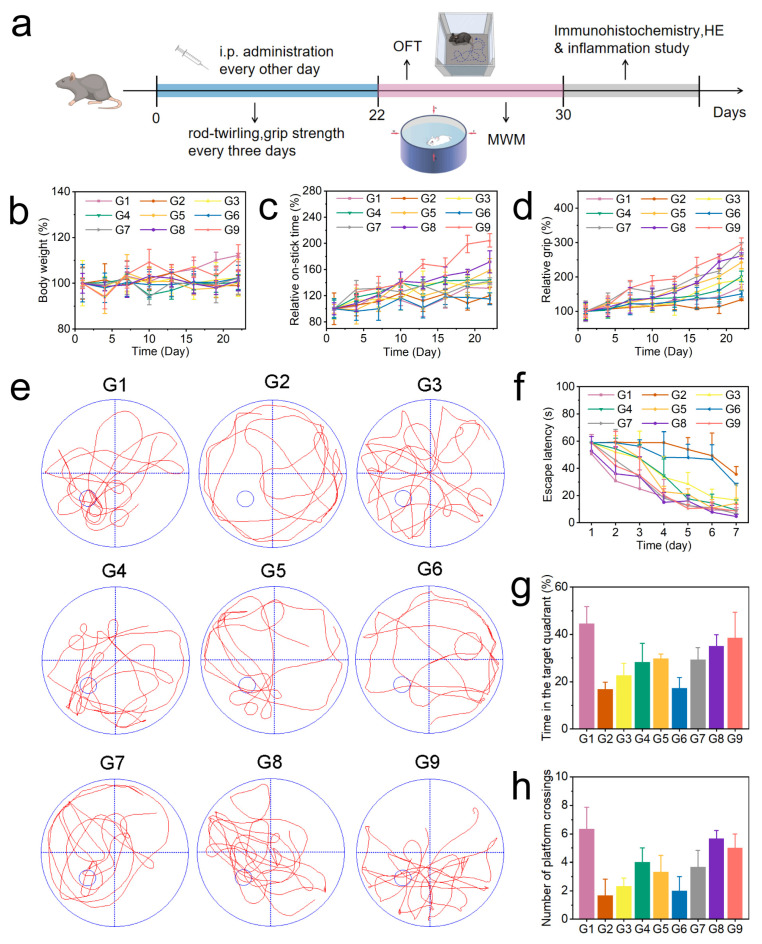
(**a**) Schedule showing the timing of the drug treatment, behavioral experiments, and therapeutic evaluation. (**b**) Changes in the body weight of mice. (**c**) Rotarod experiment. (**d**) Grip test. (**e**) Representative trajectory of mice in the water maze test. (**f**–**h**) Average escape latency, time spent in the target quadrant, and number of platforms crossings by the mice. (G1, WT; G2, AD; G3, AD + BZ; G4, AD + CUR; G5, AD + BZ + CUR; G6, AD + COF-TB; G7, AD + B@COF-TB; G8, AD + C@COF-TB; G9, AD + BC@COF-TB) (*n* = 5).

We further studied the effect of BC@COF-TB on ameliorating the cognitive impairment in 5×FAD mice. After the purchased male 5×FAD mice were acclimated to the barrier system for one week. The 5×FAD mice were randomly divided into nine groups: WT, AD, AD + BZ, AD + CUR, AD + BZ + CUR, AD + COF-TB, AD + B@COF-TB, AD + C@COF-TB, and AD + BC@COF-TB. Each group contained five mice. According to the groups, different treatments were given for three weeks, and the treatment method was intraperitoneal injection every two days. Behavioral testing was conducted according to the schedule shown in Figure 4a. The effect of COF administration on AD mice was observed.

As shown in Figure 4b, the body weight changes in mice were continuously monitored during treatment. The results showed that there was little difference in body weight changes among the different treatment groups. It was proven again that the drug delivery system had good biocompatibility. During the treatment period, the neural motor ability of mice was measured using the rotarod (Figure 4c) and grip strength experiments (Figure 4d) every three days. The results showed that compared with the AD group, the neural motor ability of the BC@COF-TB group was significantly improved. The single drug-loaded COF resulted in a certain cognitive improvement, but the therapeutic activity of the single drug-loaded COF was significantly lower than that of the double drug-loaded COF, indicating that the multi-target treatment may be more suitable for AD. After the end of the treatment process, the changes in the memory ability and spatial exploration ability of mice were verified by performing an open field test (Figure S19) and Morris water maze test (Figure 4e–h). The results were similar to those of the cognitive test. Compared with the AD group, the memory ability and spatial exploration ability of the BC@COF-TB group were significantly improved, which once again proved the effectiveness of multi-target double-drug treatment and showed the potential of the COF double-drug delivery system proposed in this study in the treatment of AD. Subsequently, all experimental mice were euthanized according to ethical requirements, and brain tissue was taken for immunohistochemical detection.

### 2.6. BC@COF-TB Allaeviates Inflammatory Injury and Aβ Deposition in AD Mice

**Figure 5:**
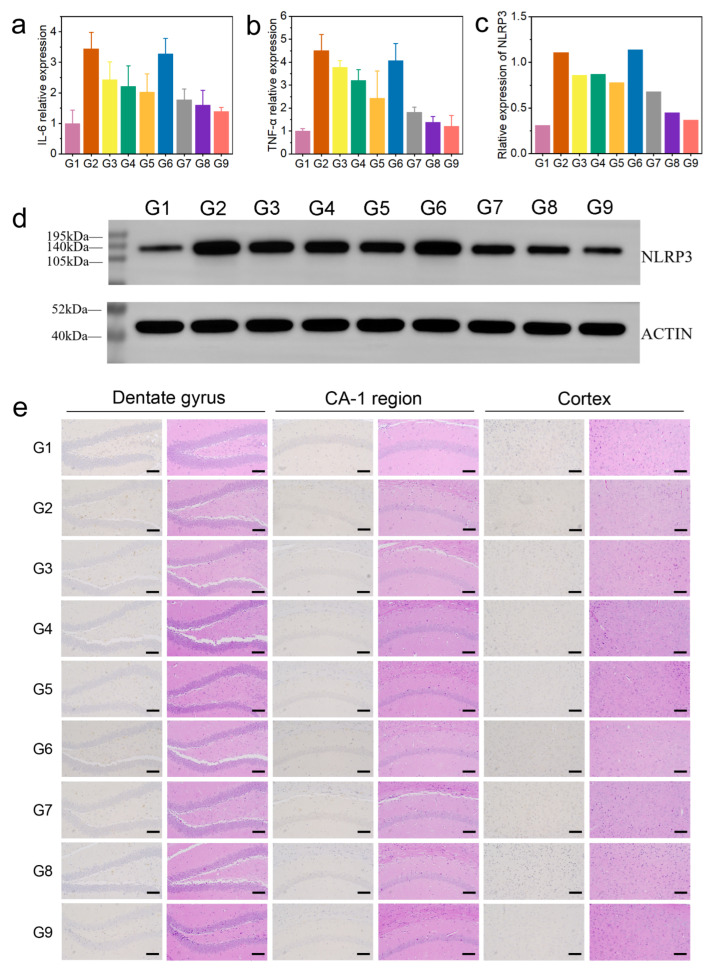
(**a**) Relative expression of IL-6. (**b**) Relative expression of TNF-α. (**c**) Quantitative analysis of WBs. (**d**) WB analysis of NLRP3 levels. (**e**) Aβ immunohistochemistry and H&E staining of the brain: hippocampal dentate gyrus, hippocampal CA-1 region, and cortex. (G1, WT; G2, AD; G3, AD + BZ; G4, AD + CUR; G5, AD + BZ + CUR; G6, AD + COF-TB; G7, AD + B@COF-TB; G8, AD + C@COF-TB; G9, AD + BC@COF-TB) (*n* = 5).

Firstly, we evaluated the levels of interleukin-6 (IL-6) and tumor necrosis factor alpha (TNF-α) in mouse brain tissue (brain tissue from euthanized mice used in animal experiments) using enzyme-linked immunosorbent assay (ELISA). Compared with the control group, the levels of IL-6 and TNF-α in the AD group were significantly increased, while BC@COF-TB reduced the levels of IL-6 and TNF-α to levels close to those in the control group (Figure 5a,b). In addition, the levels of acetylcholinesterase (AChE) and p-tau in the mouse brain were measured. Compared with the G1 group, the levels of AChE and p-tau were significantly increased in the AD group. After BC@COF-TB treatment, the levels of AChE and p-tau in the brains of AD mice were significantly reduced to levels close to those in the G1 group (Figure S20). To further investigate the anti-inflammatory effect of COF-TB, the levels of the NLRP3 inflammasome were analyzed by Western blotting (WB). The NLRP3 inflammasome is a multi-protein complex in cells that is responsible for activating the inflammatory response. The WB experimental results showed a significant downregulation of NLRP3, indicating that BC@COF-TB has strong anti-inflammatory activity (Figure 5c,d). These results indicate that CUR and BZ loaded in COF-TB have excellent anti-inflammatory effects.

The hippocampus is closely related to learning and spatial memory. To observe the deposition of Aβ in the mouse brain, we performed immunohistochemical detection (Figure 5e). There was no deposition of Aβ in the brains of WT mice, and the content of Aβ in the brains of 5×FAD mice increased significantly. The content of Aβ in the brains of mice in BZ, CUR, BZ + CUR, B@COF-TB, C@COF-TB, and BC@COF-TB groups decreased to varying degrees, among which the COF-TB treatment group had the lowest content of Aβ in the brain. COF-TB, a drug carrier, can increase therapeutic efficacy by improving drug utilization. The significant decrease in the Aβ content in the mouse brain is the result of the combined actions of BZ and CUR loaded in COF-TB. COF-TB loaded with both drugs achieved the best therapeutic effect, which may be because CUR and BZ alleviate neuroinflammation through two different pathways. This indicates that multi-target therapy may have better therapeutic effects. The above results indicate that the treatment of AD should focus more on multi-target combination therapy, which may be an effective method for treating AD.

We also performed H&E staining of the brain and main organs of the mice to assess damage in these organs. It can be seen in Figure 5e and Figure S21 that our materials did not damage the brain or main organs of the mice, proving that COF-TB has good biocompatibility as a hydrophobic drug carrier and may have broad application prospects in the future.

## 3. Discussion

With the continuous increase in the world’s elderly population, the number of people suffering from Alzheimer’s disease is increasing annually, and the exponentially growing medical expenses are a heavy burden on social and economic development [2,3]. However, the drugs currently used to treat AD have not achieved good therapeutic effects. One of the important reasons is that many excellent drugs that can be used to alleviate AD, such as curcumin, donepezil, and resveratrol, not only cannot exert their drug activity under physiological conditions in vivo but may also cause serious side effects due to their hydrophobicity. Therefore, actively developing effective drug delivery methods is crucial for AD treatment.

COF, an ordered porous material, can be designed with different functional groups for different applications to better utilize its loading function due to its excellent loading capacity and ligand tunability. Therefore, it is widely used in various fields such as drug carriers, gas adsorption, and product separation. To achieve the loading of hydrophobic drugs in this study, a series of COFs were synthesized through rational design and screened for loading two different types of hydrophobic drugs. Among them, COF-TB had the best loading of the hydrophobic drugs CUR and BZ. When both CUR and BZ were loaded simultaneously, COF-TB also exhibited an excellent loading capacity. In drug release experiments, the drug carrier COF-TB released CUR and BZ for more than 10 h and 30 h, respectively. This sustained release is expected to increase the utilization rate of the drug in vivo.

Subsequently, COF-TB was used as a hydrophobic drug carrier to observe its therapeutic effect on LPS-induced systemic inflammation in mice. The experimental results indicate that BC@COF-TB achieved the optimal anti-inflammatory effect compared to direct drug administration, demonstrating that COF-TB not only serves as an excellent hydrophobic drug carrier but also enables the sustained release of hydrophobic drugs in vivo. This sustained release capability may be the key factor contributing to the favorable therapeutic efficacy of BC@COF-TB. In the 5×FAD mouse model, BC@COF-TB also demonstrated optimal therapeutic efficacy. Compared to other treatment groups, mice in the BC@COF-TB group exhibited superior performance in the water maze, open field, rotarod, and grip strength tests. An analysis of Aβ plaques in mice brains revealed a significant reduction in plaque formation in the BC@COF-TB group. Additionally, we observed that the BC@COF-TB treatment demonstrated superior therapeutic efficacy compared to the COF-TB monotherapy. The reason may be that AD is a multifactorial disease, and the therapeutic effect of a single drug is limited. The combined treatment based on CUR’s antioxidant effect and BZ’s anti-inflammatory effect can achieve better therapeutic effects on AD. This also suggests that the treatment of AD should not focus on a specific characteristic of AD, and multi-target therapy may achieve better therapeutic effects on AD patients.

In summary, this study designed and synthesized a drug carrier capable of loading hydrophobic drugs, which can enhance the therapeutic effect of hydrophobic drugs in vivo through drug sustained release. Co-loading of two hydrophobic drugs was achieved, with good therapeutic effects on 5×FAD mice through dual-target therapy. This drug carrier, designed for the in vivo delivery of hydrophobic drugs, has broad application potential, providing enormous potential for treatment with hydrophobic drugs in vivo.

## 4. Materials and Methods

### 4.1. Materials, Measurements and Characterization

All reagents and solvents were purchased and used commercially without further purification unless otherwise stated. Powder X-ray diffraction (PXRD) data were measured from 2° to 40° using a Dmax2200PC diffractometer (Rigaku, Tokyo, Japan). TGA was performed on a METTLER-TOLEDO TGA/DSC thermogravimetric (METTLER TOLEDO, Zurich, Switzerland) analyzer in the range of 30–800 °C (air). The nitrogen adsorption–desorption isotherms of the samples were measured on a Quantachrome Autosorb-iQ2 gas adsorption instrument (Quantachrome, Boynton Beach, FL, USA) at 77K. The absorbance of the sample was measured in the range of 200–800 nm using a VARIAN Cary-60 UV-visible spectrophotometer (Agilent Technologies, Santa Clara, CA, USA). Zeta potential was measured on a Zetasizer Nano ZSE (Malvern Panalytical, Worcestershire, UK). Record scanning electron microscopy (SEM) images on Hitachi SU-8010 (Hitachi, Tokyo, Japan) devices at an acceleration voltage of 5 kV. Transmission electron microscopy (TEM) images were recorded on a JEOL 2100F (JEOL, Tokyo, Japan) device with an acceleration voltage of 300 kV. Fourier transform infrared spectroscopy (FT-IR) was collected using Nicolet IS50 FT-IR (Thermo Scientific, Waltham, MA, USA) spectrometer. Measure the absorbance using an INFINITE M NANO (Tecan, Zurich, Switzerland) enzyme-linked immunosorbent assay (ELISA) reader.

The following reagents were purchased from Merck Group Darmstadt, Germany. N-methylene bisacrylamide (99%), DPPH· (≥98.5%), ABTS· (>99%), 4,4′,4″-(1,3,5-Triazine-2,4,6-triyl) trianiline (≥95%), 4,4′,4″-(1,3,5-Triazine-2,4,6-triyl) trianiline (97%), benzene-1,3,5-tricarboxaldehyde (97%), 1,4-dioxane (99%), 4,4′,4″-(1,3,5-Triazine-2,4,6-triyl) trianiline (97%), 1,3,5-trimethylbenzene (97%), Curcumin (98%), 2,1,3-benzoxadiazole (99.7%). The following reagents were purchased from Wuhan Servicebio Biotechnology Co., Ltd., Wuhan, China. ACTIN (Rabbit), NLPR3(Rabbit), fluorescent secondary antibody (goat), PVDF membrane (0.45 μm), BCA protein quantitative detection kit, 5 × SDS-PAGE protein loading buffer, Rapid preparation of reagent kit, Protein-free rapid blocking solution, TBST buffer solution, PMSF, RIPA cracking solution, BS, DMEM. The nitric oxide detection kit was purchased from Shanghai Biyuntian Biotechnology Co., Ltd., Shanghai, China. The mouse TNF-α detection kit and mouse IL-6 detection kit were purchased from Shanghai Enzyme linked Biotechnology Co., Ltd., Shanghai, China. Tetrahydrofuran (>99%), acetic acid (>99%), methanol (>99%), ethanol (>99%), and Sodium hydroxide (>99%) were all purchased from China National Pharmaceutical Group Chemical Reagent Co., Ltd., Shanghai, China.

### 4.2. Synthesis of COFs

Synthesis of COF-TB: A mixture of 4,4′,4″-(1,3,5-Triazine-2,4,6-triyl) trianiline (TTA, 0.2 mmol, 70.884 mg), benzene-1,3,5-tricarboxaldehyde (TB, 0.2 mmol, 32.428 mg), 1,4-dioxane (0.69 mL), 1,3,5-trimethylbenzene (4.11 mL) and acetic acid (3 M, 0.8 mL) was charged in a Pyrex tube. Subsequently, the tube was heated at 120 °C for 120 h. The resulting precipitate was filtered, and washed with tetrahydrofuran to remove the trapped guest molecules. The powder was collected and dried under vacuum conditions at 80 °C for 12 h to yield COF-TB as a yellow powder (yield 80.18 ± 1.12%).

Synthesis of COF-TP: A mixture of 4,4′,4″-(1,3,5-Triazine-2,4,6-triyl) trianiline (TTA, 0.2 mmol, 70.884 mg), 2,4,6-triformylphloroglucinol (TP, 0.08 mmol, 16.811 mg), 1,4-dioxane (1.5 mL), 1,3,5-trimethylbenzene (1.5 mL) and acetic acid (6 M, 0.3 mL) was charged in a Pyrex tube. Subsequently, the tube was heated at 120 °C for 72 h. The resulting precipitate was filtered and washed with tetrahydrofuran to remove the trapped guest molecules. The powder was collected and dried under vacuum conditions at 80 °C for 12 h to yield COF-TP as a yellow powder (yield 85.08 ± 1.75%).

Synthesis of COF-TC: A mixture of 4,4′,4″-(1,3,5-Triazine-2,4,6-triyl) trianiline (TTA, 0.2 mmol, 70.884 mg), 2,4,6-trimethylbenzene-1,3,5-tricarbaldehyde (TC, 0.2 mmol, 40.884 mg), 1,4-dioxane (0.69 mL), 1,3,5-trimethylbenzene (4.11 mL) and acetic acid (3 M, 0.8 mL) was charged in a Pyrex tube. Subsequently, the tube was heated at 120 °C for 120 h. The resulting precipitate was filtered, and washed with tetrahydrofuran to remove the trapped guest molecules. The powder was collected and dried under vacuum condition at 80 °C for 12 h to yield COF-TC as a yellow powder (yield 67.37 ± 2.61%).

### 4.3. Drug Loading into COFs

Synthesis of B@COF-TB, B@COF-TP, B@COF-TC: The COF-TB/COF-TP/COF-TC (40 mg) and BZ (120 mg) were dissolved in anhydrous ethanol (40 mL) and stirred at room temperature for 24 h. After filtration and drying, B@COF-TB/B@COF-TP/B@COF-TC was obtained.

Synthesis of C@COF-TB, C@COF-TP, C@COF-TC: The COF-TB/COF-TP/COF-TC (40 mg) and CUR (100 mg) were dissolved in anhydrous ethanol (40 mL) and stirred at room temperature for 24 h. After filtration and drying, C@COF-TB/C@COF-TP/C@COF-TC was obtained.

Synthesis of BC@COF-TB, BC@COF-TP, BC@COF-TC: Add COF-TB, COF-TP and BC@COF-TC separately to a mixed solution of BZ (120 mg) and CUR (100 mg) in anhydrous ethanol (40 mL) and stir at room temperature for 24 h. After filtration and drying, it can be obtained BC@COF-TB, BC@COF-TP and BC@COF-TC.

### 4.4. Detection of Nitric Oxide

Nitric oxide production was detected by the Griess kit. The released nitric oxide is easily converted into nitrite, which reacts with Griess reagent and has a UV absorption peak at 540 nm. The UV absorbance at 540 nm was measured by a microplate reader, and the concentration of nitric oxide was obtained by a NaNO_2_ standard curve.

### 4.5. Detection of CUR

CUR was dissolved in anhydrous ethanol, and different concentration gradients of 1–10 μg/mL were prepared. The absorbance at 426 nm was detected by a ultraviolet-visible spectrophotometer, and the standard curve was made. The concentration of CUR can be obtained by substituting the absorbance of CUR in the test sample into the standard curve.

### 4.6. Determination of Drug Loading

#### 4.6.1. Determination of BZ Loading in B@COF-TB, B@COF-TP, B@COF-TC, BC@COF-TB, BC@COF-TP, BC@COF-TC

The 2 mg B@COF-TB was dispersed in 18 mL PBS solution containing 0.5 mM N-acetylcysteine (NAC) and incubated in a shaker at 37 °C for 120 h. The concentration of nitric oxide was detected by the Griess kit, and the content of BZ was calculated. Drug Loading efficiency (DLC%) = 100% × weight of BZ/weight of B@COF-TB. The calculation method for the loading efficiency of B@COF-TP, B@COF-TC, BC@COF-TB, BC@COF-TP, BC@COF-TC is similar to the above method.

#### 4.6.2. Determination of CUR Loading in C@COF-TB, C@COF-TP, C@COF-TC, BC@COF-TB, BC@COF-TP, BC@COF-TC

The 10 mg C@COF-TB was dispersed in 40 mL of anhydrous ethanol, and the material was completely broken by a cell crusher, so that CUR was completely released. The supernatant was obtained by centrifugation, and the concentration of CUR was measured by an ultraviolet-visible spectrophotometer and a standard curve. Drug Loading efficiency (DLC%) = 100% × weight of CUR/weight of C@COF-TB. The calculation method for the loading efficiency of B@COF-TP, B@COF-TC, BC@COF-TB, BC@COF-TP, BC@COF-TC is similar to the above method. When measuring the loading efficiency of BC@COF-TB, BC@COF-TP, and BC@COF-TC, an additional 0.5 m of 0.5 mM N-acetylcysteine needs to be added to the system.

### 4.7. Study on Drug Release In Vitro

In vitro release of nitric oxide: 2 mg B@COF-TB/B@COF-TP/B@COF-TC/BC@COF-TB/BC@COF-TP/BC@COF-TC was dispersed in 10 mL PBS solution containing 0.25 mM N-acetylcysteine and incubated in a 37 °C shaker. Remove 0.2 mL of solution at regular intervals. The absorbance at 540 nm was read by a microplate reader and substituted into the standard curve to obtain the release concentration of nitric oxide.

In vitro release of CUR: 10 mg drug drug-loaded COF were dispersed in 40 mL PBS (pH = 7.4, 20% Tween 20) and incubated on a 37 °C shaker. Remove 1 mL of the solution at regular intervals and replenish the same amount of fresh PBS solution. The absorbance at 425 nm was read by a ultraviolet-visible spectrophotometer, and the release concentration of CUR was obtained by substituting it into the standard curve.

The release percentage of CUR = the release amount of CUR/the total amount of CUR in the material × 100%.

### 4.8. Antioxidant Activity Test

DPPH· scavenging experiment: The sample solution (4.6 mL) was mixed with DPPH· ethanol solution (1 mM, 0.4 mL), and the mixture was placed in the dark at room temperature to react. The absorbance was measured at 517 nm after 30 min. The DPPH· scavenging rate was calculated according to the formula:

DPPH· scavenging rate (%) = (1 − A/A0) × 100%

A = sample group solution absorbance.
A0 = blank group solution absorbance.

ABTS·+ scavenging experiment: The equal volume of ABTS solution (7 mM) and (NH_4_)_2_S_2_O_8_ solution (2.45 mM) was mixed at room temperature and incubated in the dark for 12–16 h to generate ABTS free radicals. Then, an appropriate amount of ethanol was added until the absorbance of the solution was about 0.70 at 734 nm. Take 3.9 mL ABTS·+ solution, add 0.1 mL sample solution, mix well, react in the dark at room temperature for 6 min, and determine the absorbance at 734 nm. The ABTS· scavenging rate was calculated according to the formula:

ABTS·+ scavenging rate (%) = (1 − A/A0) × 100%

A = sample group solution absorbance.
A0 = blank group solution absorbance.

### 4.9. LPS-Induced Inflammation Model Anti-Inflammatory Experiments

ICR mice (male, 6–8 weeks) were purchased from Changchun Yisi Experimental Animal Technology Co., Ltd., Changchun, China. Animals were fed in groups (3 mice/cage). All mice were kept in an environment with a temperature of (24 ± 2 °C) and a humidity of (60 ± 5%) for a 12 h light/dark cycle and were randomly provided with food and water. The ICR mice were divided into 9 groups, 3 in each group. The control group was intraperitoneally injected with 100 μL normal saline, and the other eight groups were intraperitoneally injected with 100 μL LPS (1 mg/kg) every day for five days. BZ, CUR, BZ + CUR, COF-TB, B@COF-TB, C@COF-TB and BC@COF-TB (dose BC@COF-TB = 10 mg/kg) were injected into each group from the sixth day. During the period, the weight changes in mice were detected. After all data collection is completed, euthanize all mice. Collect mouse blood samples and measure the level of IL-6 in serum through ELISA analysis.

### 4.10. Hemolysis Experiment

The fresh blood of mice was centrifuged to obtain red blood cells. 0.5 mL of different concentrations of COF-TB or BC@COF-TB (10, 50, 100, 200, 500 μg/mL) was mixed with 0.5 mL of red blood cells in a test tube. Pure water was used as a positive control and PBS was used as a negative control. The UV absorbance at 541 nm was measured by a ultraviolet-visible spectrophotometer after centrifugation of the mixture.

### 4.11. Animal Experiment

The mice (5×FAD, C57BL/6) were from Gempharmatech Co., Ltd., Nanjing, China. (male, 8 months). Animals were fed in groups (5 mice/cage). All mice were kept in an environment with a temperature of (24 ± 2 °C) and a humidity of (60 ± 5%) for a 12 h light/dark cycle, and were randomly provided with food and water. All animal experiments were carried out in accordance with the principles and procedures of the “Regulations on the Administration of Laboratory Animals” approved by the State Council on 31 October 1988, and the “Regulations on the Administration of the Storage and Use of Laboratory Animals in China” promulgated by the National Science and Technology Commission on 14 November 1988. All animal experiments were supervised by the Animal Health and Use Committee of Northeast Normal University. Authorization Number: 202502059. Approval date: 1 January 2025.

Conduct all animal experiments according to the time shown in Figure 4a. After all data collection is completed, euthanize all mice. Extract the mouse brain tissue and major organs for subsequent analysis.

#### 4.11.1. Grouping Situation

The control group consisted of five wild-type (WT) mice. The experimental groups were AD group, AD + BZ group, AD + CUR group, AD + BZ + CUR group, AD + COF-TB group, AD + B@COF-TB group, AD + C@COF-TB group, AD + BC@COF-TB group, five mice in each group. The dose was based on BC@COF-TB = 10 mg/kg. Intraperitoneal injection (100 μL/time) was performed every other day for a total of 21 days and 11 times. The body weight of the mice was monitored every three days during the administration period, and the rod-twirling and forelimb tension tests were performed for a total of 22 days.

#### 4.11.2. Behavioral Tests

Rod-twirling experiment: The instrument used in the experiment is the ZH-600 B rotating rod fatigue tester (Huaibei Zhenghua Biological Instruments Co., Ltd., Suixi, China). The diameter of the rotating rod is about 3 cm, and the width of the channel is about 8 cm. The mice were prevented from the rotating rod, and the rotating rod was accelerated from 0 to 50 rpm. After placing the mice, start accelerating and record the time they stay on the spinning stick and the speed at which they fall.

Grip test: Place the forelimb of the mice on the tension meter, gently pull the tail of the mice, so that the mice grasps the iron mesh, gradually increase the force until the mice is separated from the tension meter and record the tension reading.

Open field test: The mice were placed separately in a 60 cm × 60 cm × 40 cm box and allowed to move freely for 2 min to adapt to the environment. Subsequently, their trajectories were recorded for 5 min while the surrounding environment remained dark and quiet.

Morris water maze experiment: The device consists of a circular bathtub and a camera. The bathtub is divided into four quadrants. The mice enter from the first quadrant, and the target quadrant is defined as the third quadrant. The mice were trained for six consecutive days to find a platform in the middle of the third quadrant. On the sixth day, the platform was removed, and the trajectory of the mice within one minute, the residence time in the target quadrant and the number of times crossing the platform were recorded.

### 4.12. Western Blotting Analysis

Wash a portion of the brain tissue with pre cooled PBS 2–3 times to remove blood stains, cut it into small pieces and place them in a homogenate tube. Add 4 mm homogenization beads and 10 times the volume of tissue lysate (Servicebio, G2002). Set up a homogenization program for homogenization. Afterwards, the brain homogenate was centrifuged at 12,000 rpm and 4 °C for 10 min, and the supernatant was collected to obtain the total protein solution. Measure the protein concentration in the supernatant using the BCA protein concentration kit (Servicebio, G2026) and dilute the protein concentration to the same level. Add the diluted protein solution (Servicebio, G3375) to the reduced protein loading buffer in a 4:1 ratio, denature in a boiling water bath for 15 min, and store at −20 °C for future use. The NLRP3 (Servicebio, GB114320) was separated by SDS-PAGE gel electrophoresis and transferred to PVDF membrane (Servicebio, G6047). The membrane is incubated with the first antibody and then incubated with the second antibody (Servicebio, GB23303). Finally, enhanced chemiluminescence (ECL) was used to detect protein levels (Servicebio, SCG-W3000) [61].

### 4.13. Immunohistochemistry

After treating the mice brain with tissue fixative (Servicebio, G1101), it was embedded in paraffin. The paraffin blocks were cut into 4 μm sections, dewaxed with xylene, and rehydrated with ethanol. After 1 h of blocking, the brain tissue was incubated with anti-Aβ antibody (Servicebio, GB115755, 1:200) at 4 °C overnight. Then, incubate the slices with HRP conjugated secondary antibody (Servicebio, G1301) at ambient temperature for 30 min, followed by washing with PBS. The sections were treated with DAB agent (Servicebio, G1212) to observe the immune complexes. Then, use hematoxylin to counterstain (Servicebio, G1004). Immunohistochemical staining was observed under an optical microscope (Sunny, ICX41, Yuyao, China) [62].

## 5. Conclusions

In summary, we have synthesized three imine-based COF drug delivery systems containing different modified groups, which can be used to effectively deliver hydrophobic drugs through rational design. However, COF-TB’s ability to load BZ alone is not as good as that of COF-TP. Overall, COF-TB has a better dual loading capacity for CUR and BZ than COF-TP and COF-TC. Therefore, COF-TB was chosen as the drug carrier in subsequent in vivo treatments. In the treatment of AD mice, the therapeutic effect of the COF-TB dual-drug delivery system was significantly stronger than that of a single drug treatment. This proves that the effective loading and delivery of hydrophobic drugs can play a more effective role in disease treatment. Moreover, it has been proven that the treatment of AD should actively focus on multi-target treatment methods, which are more likely to achieve good therapeutic effects. Overall, COF-TB, a material with good biocompatibility, has a high hydrophobic drug loading capacity and is expected to have greater potential for the delivery of hydrophobic drugs in vivo.

## Data Availability

The original contributions presented in this study are included in the article: Further inquiries can be directed to the corresponding author.

## Supplementary Materials

The following supporting information can be downloaded at: https://www.mdpi.com/article/10.3390/ijms262110803/s1.

## Abbreviations

The following abbreviations are used in this manuscript:

AD	Alzheimer’s disease
COFs	covalent organic frameworks
CUR	curcumin
BZ	benzofuroxan
TTA	Disperse 4,4′,4″-(1,3,5-Triazine-2,4,6-triyl) trianiline
TB	benzene-1,3,5-tricarboxaldehyde
TP	2,4,6-triformylphloroglucinol
TC	2,4,6-trimethylbenzene-1,3,5-tricarbaldehyde
TGA	thermogravimetric analysis
IL-6	interleukin-6
LPS	lipopolysaccharide
ELISA	enzyme-linked immunosorbent assay
TNF-α	tumor necrosis factor-α
AChE	acetylcholinesterase
