# Supplementary material for: Hydrophobic Drug Delivery Platforms Based on Covalent Organic Frameworks for Combined Treatment of Alzheimer’s Disease
Source: Int J Mol Sci. 2025 Nov 6;26(21):10803. doi: 10.3390/ijms262110803 (PMC12609929; doi:10.3390/ijms262110803)
Supplement: Supplementary file 1 [file ijms-26-10803-s001.zip › ijms-3918321-supplementary.pdf]

Supporting Information for

Hydrophobic drug delivery platforms based on covalent organic frameworks for combined treatment of Alzheimer's disease

Yun Zhao [1,†], Ziwei Wang [1,†], Enpeng Xi [1], Fuming Yang [1] and Nan Gao [1,*]

[1] *Key Laboratory of Polyoxometalate and Reticular Material Chemistry of Ministry of Education and Faculty of Chemistry, Northeast Normal University, Changchun 130024, P. R. China*

[*] *Correspondence: gaon320@nenu.edu.cn*

[†] *These authors contributed equally to this work.*

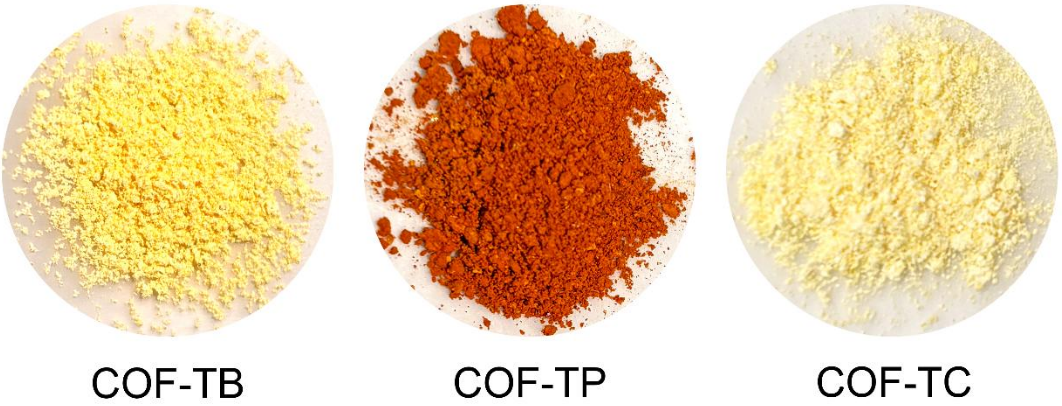

Figure S1. Image of COF-TB, COF-TP and COF-TC.

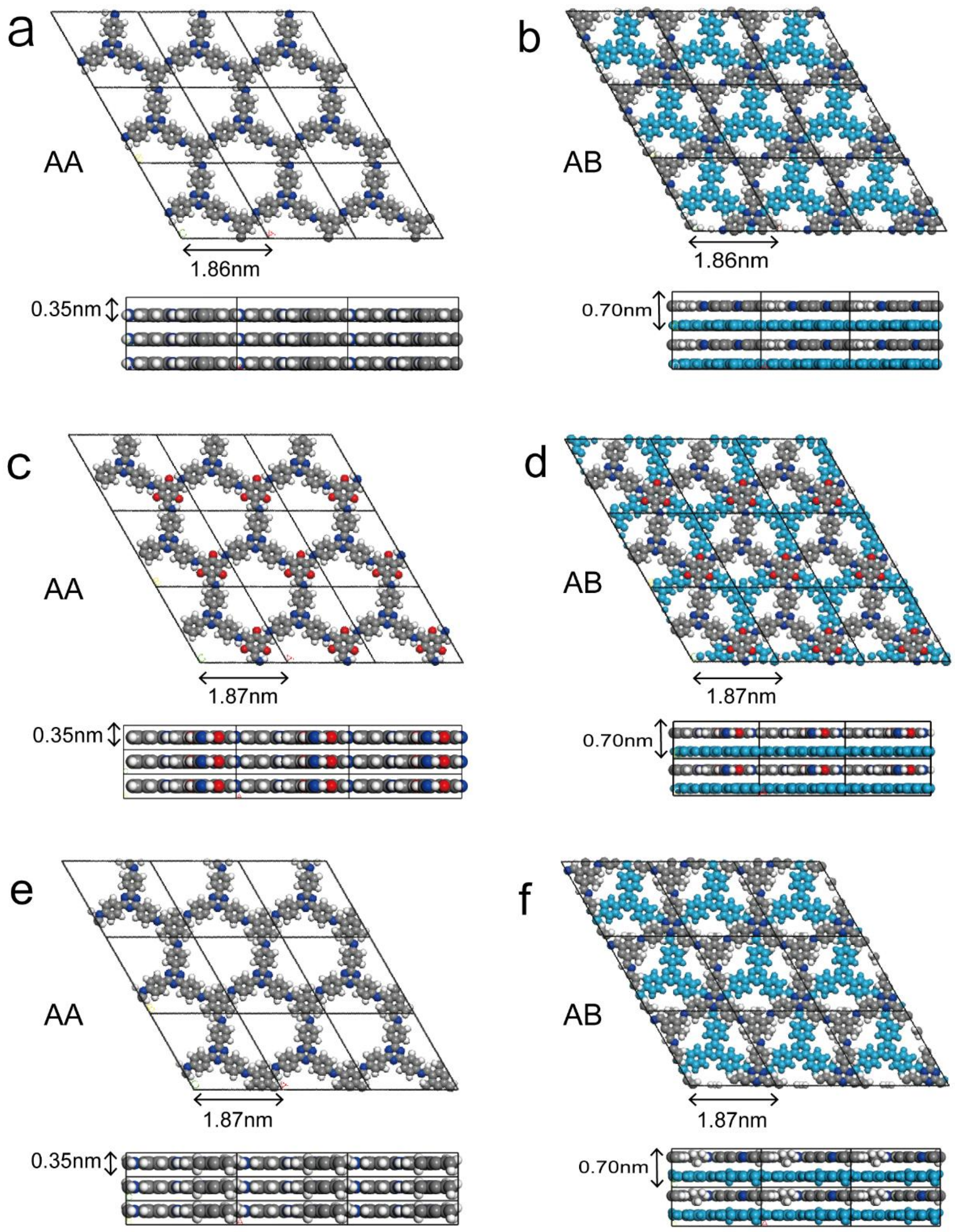

Figure S2. Simulates the stacking structure: (a) AA stacking of COF-TB. (b) AB stacking of COF-TB. (c) AA stacking of COF-TP. (d) AB stacking of COF-TP. (e) AA stacking of COF-TC. (f) AB stacking of COF-TC.

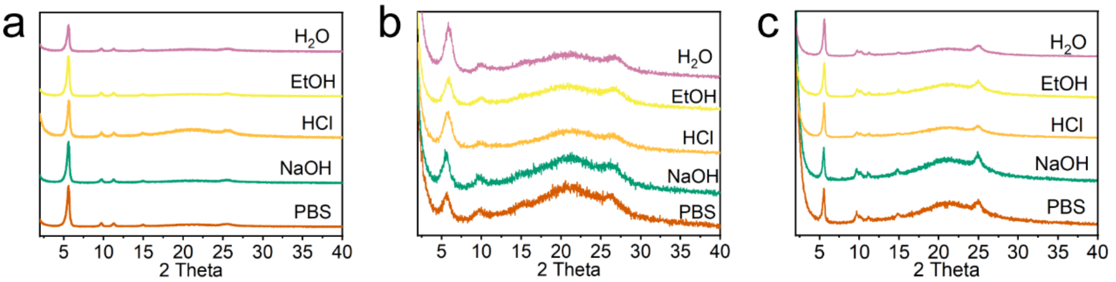

Figure S3. PXRD images of COF-TB (a), COF-TP (b) and COF-TC (c) after different solvent treatments.

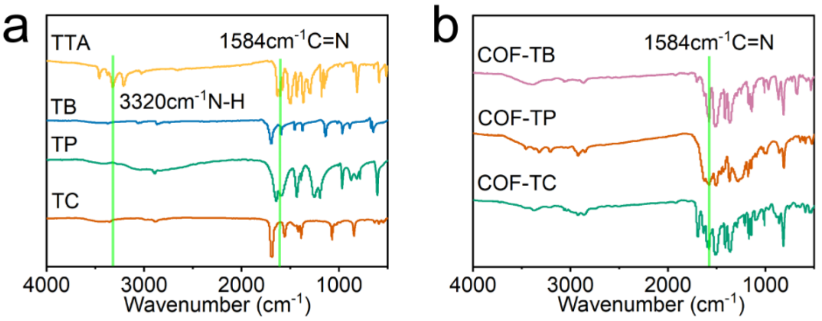

Figure S4. FT-IR images of COF-TB, COF-TP and COF-TC.

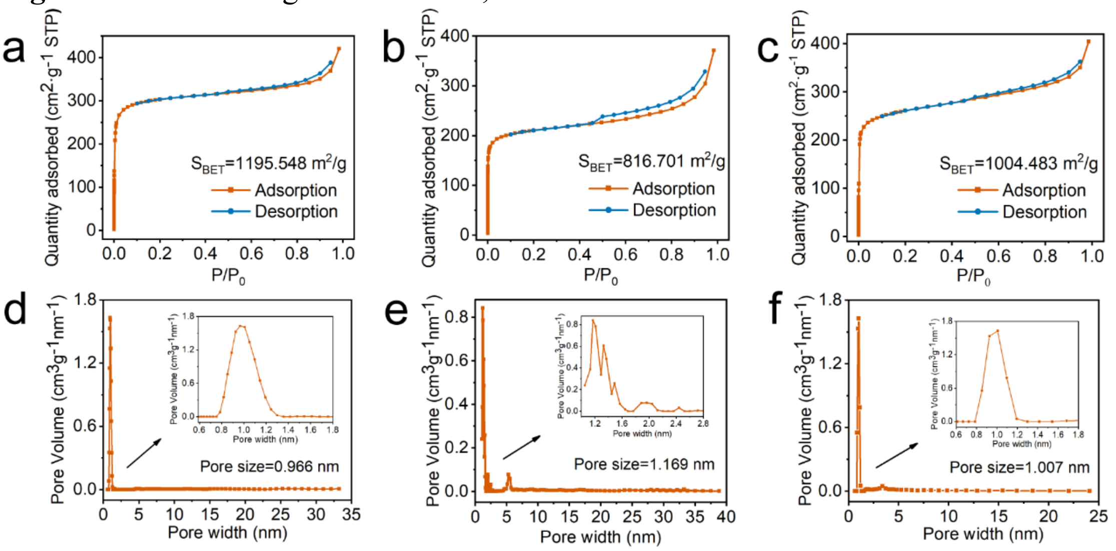

Figure S5. (a) N_2 adsorption-desorption isotherms of COF-TB. (b) N_2 adsorption-desorption isotherms of COF-TP. (c) N_2 adsorption-desorption isotherms of COF-TC. (d) Pore size distribution of COF-TB. (e) Pore size distribution of COF-TP. (f) Pore size distribution of COF-TC.

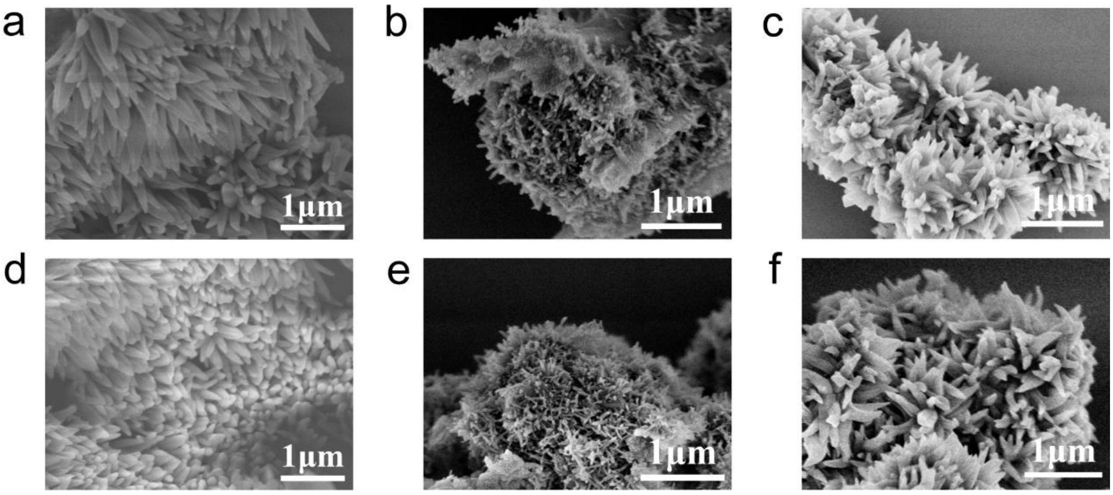

Figure S6. (a-c) SEM images of B@COF-TB, B@COF-TP and B@COF-TC. (d-f) SEM images of C@COF-TB, C@COF-TP and C@COF-TC.

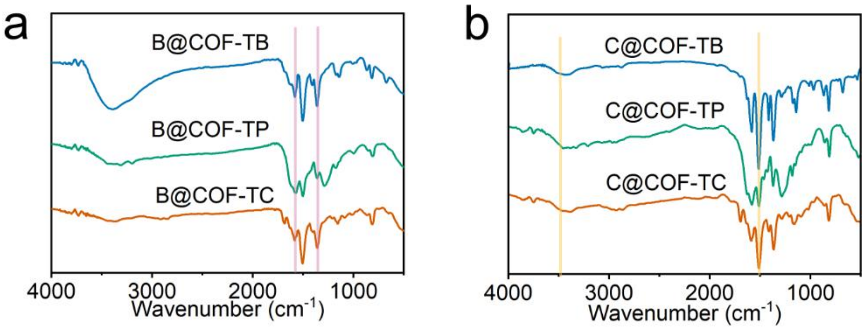

Figure S7. (a) FT-IR spectra of B@COF-TB, B@COF-TP and B@COF-TC. (b) FT-IR spectra of C@COF-TB, C@COF-TP and C@COF-TC (Purple line: the characteristic peak position of BZ, yellow line: the characteristic peak position of CUR).

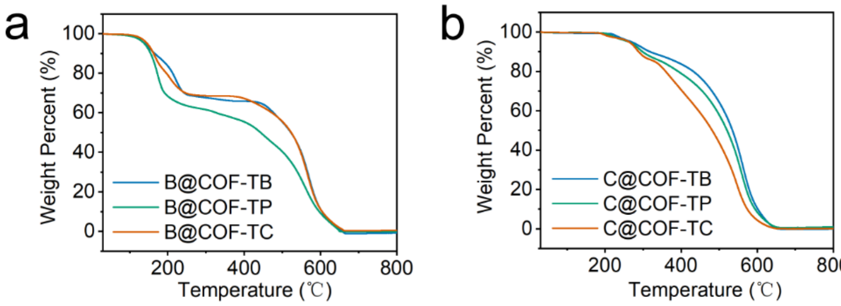

Figure S8. (a) TGA of B@COF-TB, B@COF-TP and B@COF-TC. (b) TGA of C@COF-TB, C@COF-TP and C@COF-TC.

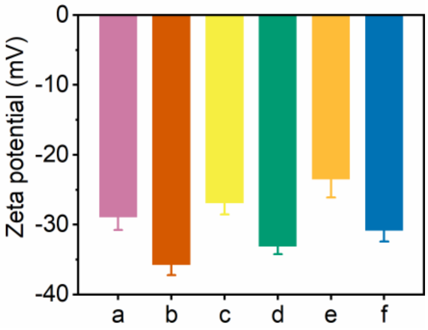

Figure S9. Zeta potential diagram. (a: B@COF-TB, b: C@COF-TB, c: B@COF-TP, d:

C@COF-TP, e: B@COF-TC, f: C@COF-TC)

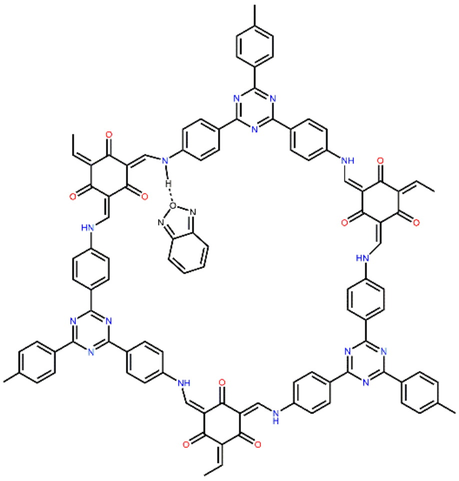

Figure S10. Schematic diagram of hydrogen bonding between COF-TC and BZ.

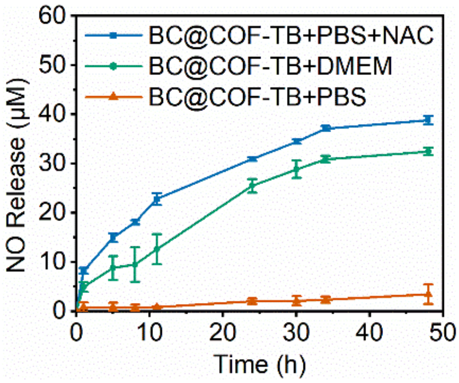

Figure S11. NO release curve of BC@COF-TB in PBS + NAC, DMEM and PBS solutions. (NAC: N-acetylcysteine)

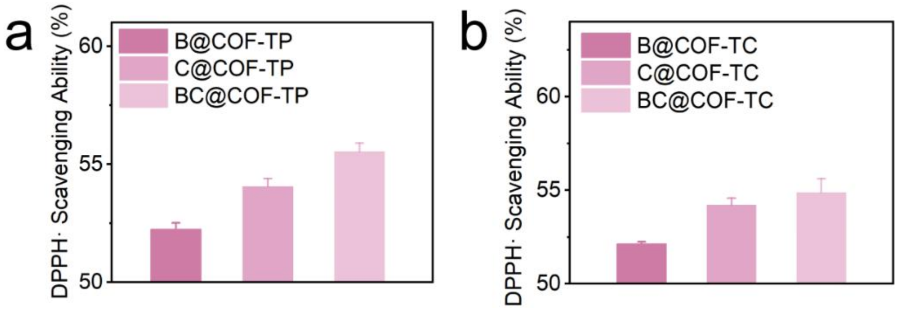

Figure S12. (a) DPPH· scavenging experiments of B@COF-TP, C@COF-TP, BC@COF-TP. (b) DPPH· scavenging experiments of B@COF-TC, C@COF-TC, BC@COF-TC.

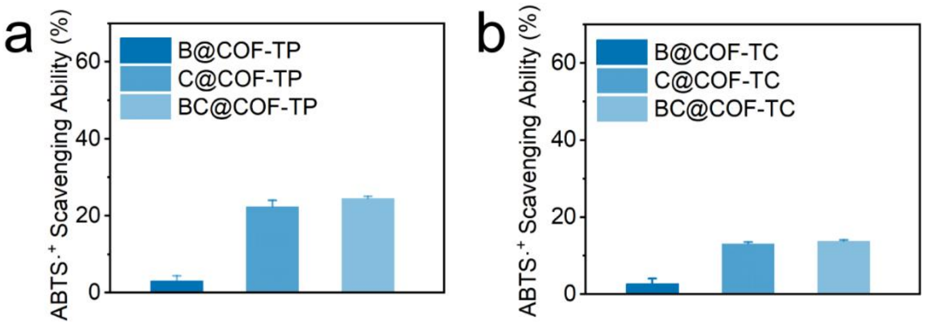

Figure S13. (a) ABTS$\cdot^{+}$ scavenging experiments of B@COF-TP, C@COF-TP and BC@COF-TP. (b) ABTS$\cdot^{+}$ scavenging experiments of B@COF-TC, C@COF-TC and BC@COF-TC.

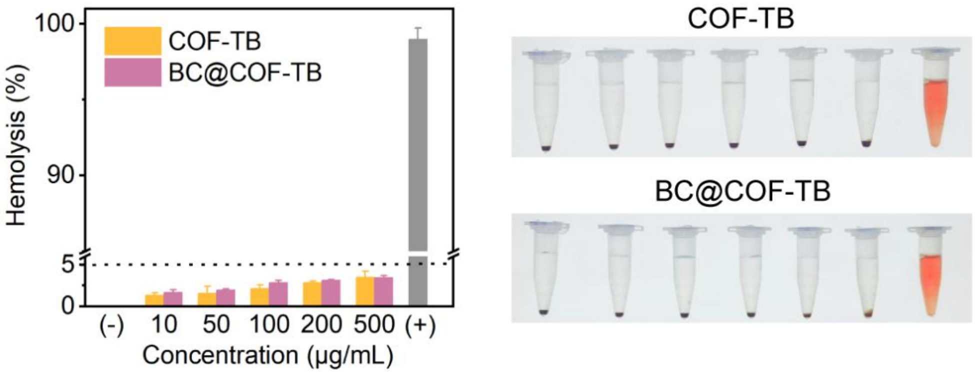

Figure S14. Hemolysis test of red blood cells in PBS, different concentrations of COF-TB and BC@COF-TB, deionized water (DI). And the corresponding digital photograph.

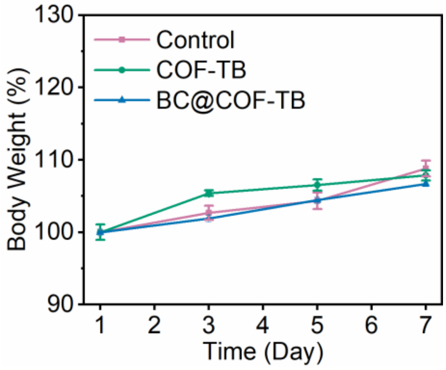

Figure S15. Changes in body weight of mice after treatment with different materials.

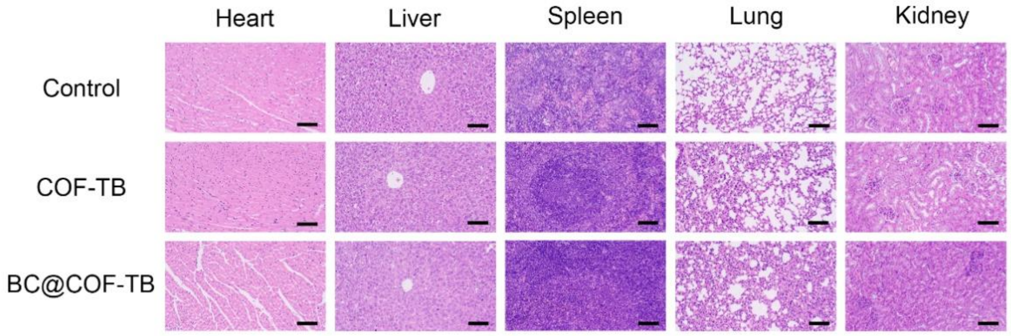

Figure S16. H&E staining of heart, liver, spleen, lung and kidney. (Scale bar: 200 μm).

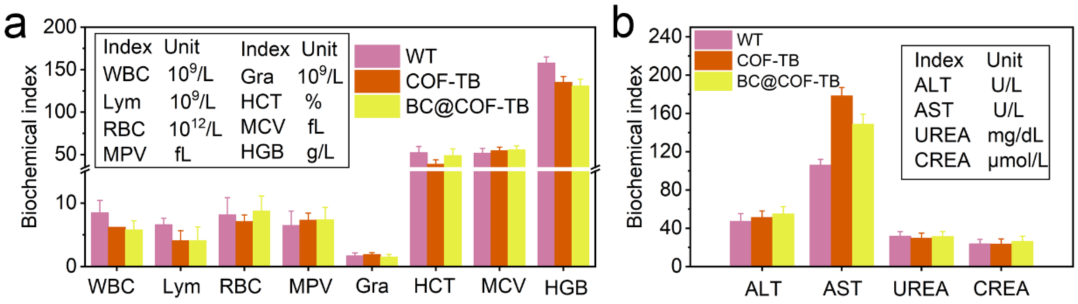

Figure S17. COF-TB biocompatibility testing. Blood routine (a) and blood (b) biochemistry indexes.

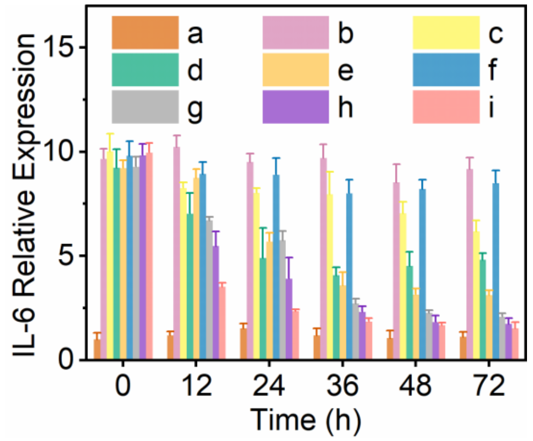

Figure S18. IL-6 relative expression content (a: control group, b: LPS, c: LPS + BZ, d: LPS + CUR, e: LPS + BZ + CUR, f: LPS + COF-TB, g: LPS + B@COF-TB, h: LPS + C@COF-TB, i: LPS + BC@COF-TB).

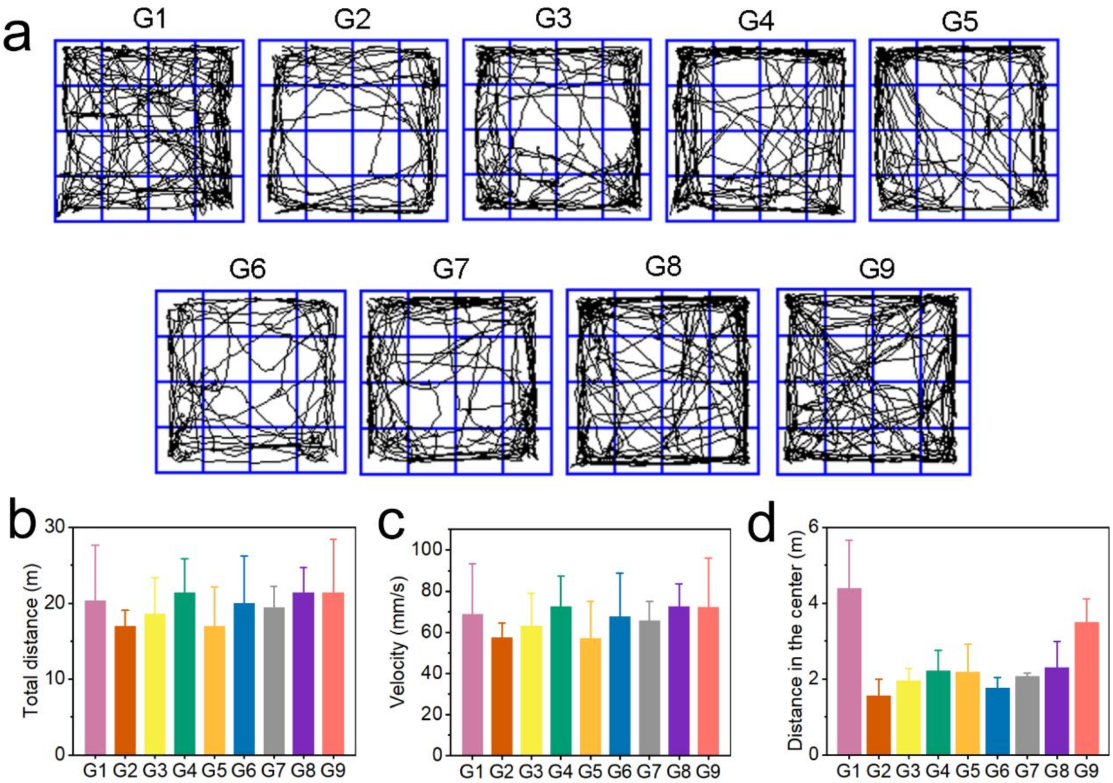

Figure S19. (a) Movement track of mice in open field. (b-d) Total distance, velocity and distance in the center of mice in open field. (G1: WT, G2: AD, G3: AD + BZ, G4: AD + CUR, G5: AD + BZ + CUR, G6: AD + COF-TB, G7: AD + B@COF-TB, G8: AD + C@COF-TB, G9: AD + BC@COF-TB)

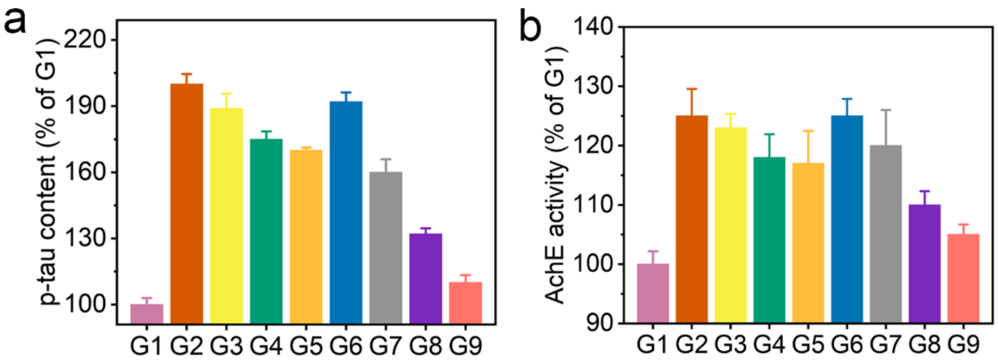

Figure S20. The levels of AChE and p-tau in the brains of mice after different treatments. (G1: WT, G2: AD, G3: AD + BZ, G4: AD + CUR, G5: AD + BZ + CUR, G6: AD + COF-TB, G7: AD + B@COF-TB, G8: AD + C@COF-TB, G9: AD + BC@COF-TB)

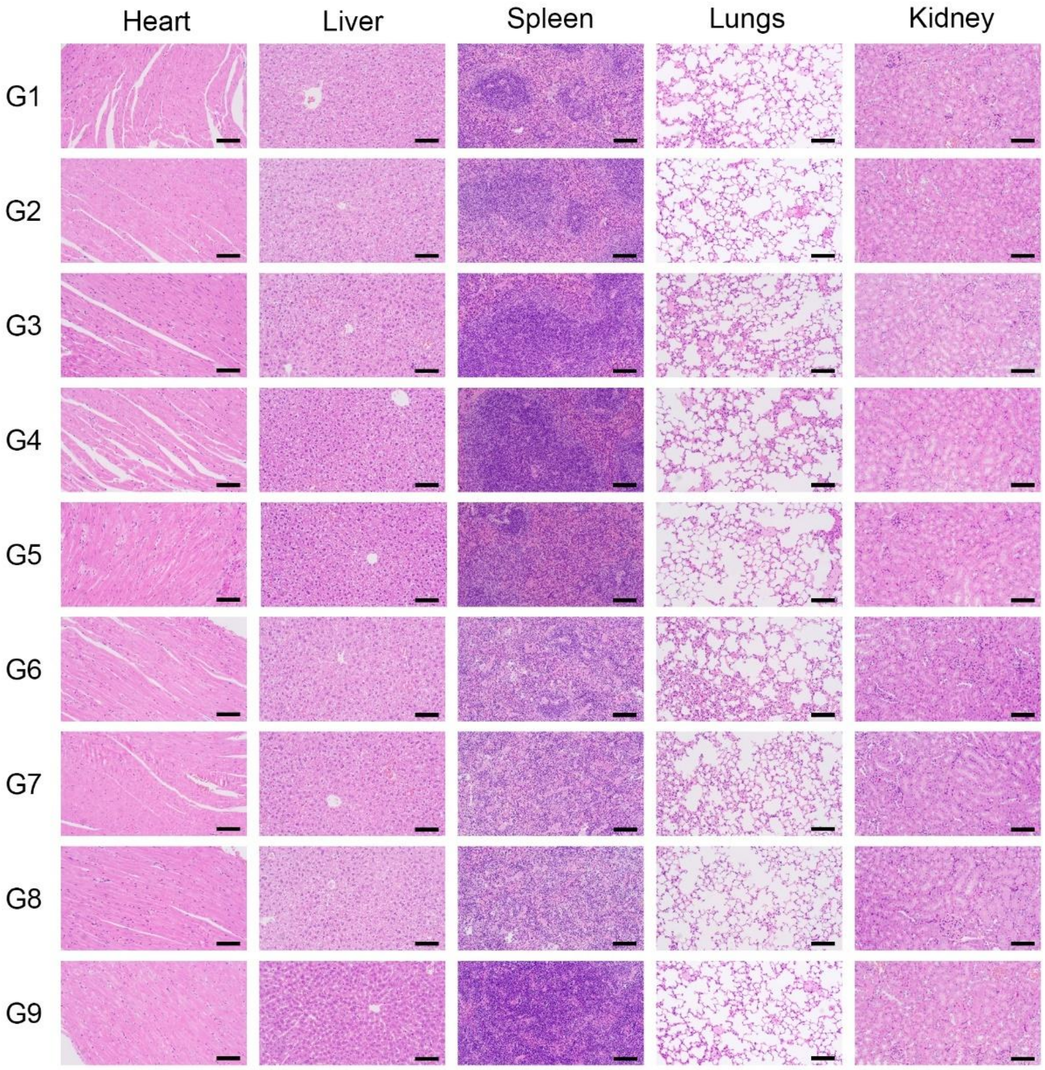

Figure S21. H&E staining of heart, liver, spleen, lung and kidney. (Scale bar: 200 μm). （G1: WT, G2: AD, G3: AD+BZ, G4: AD+CUR, G5: AD+BZ+CUR, G6: AD+COF-TB, G7: AD+B@COF-TB, G8: AD+C@COF-TB, G9: AD+BC@COF-TB).

Table S1. Fractional atomic coordinates for the unit cell of COF-TB.
AA stacking of COF-TB:

COF-TB: space group symmetry: P1<br>a = b = 18.5576 Å c = 3.5065 Å<br>α = β = 90 ° γ = 120 °							
Atom	x	y	z	Atom	x	y	z
C1	0.57871	0.83896	0.25885	C28	0.71196	0.15866	0.25887
C2	0.52908	0.75236	0.25885	C29	0.74595	0.10636	0.25887
C3	0.65263	0.73975	0.25886	C30	0.83257	0.14096	0.25887
C4	0.70232	0.82637	0.25886	C31	0.88487	0.22725	0.25887
N5	0.54893	0.56216	0.25887	C32	0.85027	0.27927	0.25887
C6	0.51333	0.61075	0.25886	N33	0.71846	0.96501	0.25886

N7	0.42907	0.57443	0.25886	C34	0.68994	1.01608	0.25886
C8	0.38048	0.49024	0.25887	C35	0.97515	1.26152	0.25887
N9	0.4168	0.4423	0.25887	N36	1.02622	1.34111	0.25886
C10	0.50099	0.47791	0.25887	H37	0.54826	0.87921	0.25885
C11	0.54	0.42557	0.25888	H38	0.4584	0.7222	0.25885
C12	0.28914	0.45123	0.25887	H39	0.68325	0.69964	0.25886
C13	0.56567	0.70209	0.25886	H40	0.773	0.85651	0.25887
C14	0.49068	0.3386	0.25889	H41	0.41996	0.30798	0.2589
C15	0.52762	0.28891	0.25889	H42	0.48708	0.21824	0.2589
C16	0.61423	0.32541	0.25888	H43	0.73452	0.44297	0.25886
C17	0.66382	0.41252	0.25887	H44	0.66737	0.53283	0.25886
C18	0.62685	0.46215	0.25887	H45	0.29159	0.57127	0.25882
C19	0.25149	0.50055	0.25884	H46	0.13472	0.50415	0.25881
C20	0.16486	0.46361	0.25884	H47	0.11202	0.25671	0.25891
C21	0.11475	0.377	0.25886	H48	0.26904	0.32386	0.25891
C22	0.15227	0.32742	0.25889	H49	0.77463	0.37129	0.25887
C23	0.23887	0.36438	0.25889	H50	0.64155	0.13093	0.25887
C24	0.66582	0.87648	0.25886	H51	0.86071	0.09864	0.25887
N25	0.65013	0.27277	0.25888	H52	0.89259	0.34973	0.25887
C26	0.72971	0.30129	0.25887	H53	0.61969	0.98941	0.25886
C27	0.76398	0.24528	0.25887	H54	1.00182	1.21793	0.25888

AB stacking of COF-TB:

COF-TB: space group symmetry: P1<br>a=b= 18.5576Å c=7.0135Å<br>α=β=90° γ=120°							
C1	0.57871	0.83896	0.12943	C55	0.58856	0.17372	0.62942
C2	0.52908	0.75236	0.12943	C56	0.50194	0.13678	0.62942
C3	0.65263	0.73975	0.12943	C57	0.45182	0.05017	0.62943
C4	0.70232	0.82637	0.12943	C58	0.48934	0.00059	0.62944
N5	0.54893	0.56216	0.12943	C59	0.57594	0.03755	0.62944
C6	0.51333	0.61075	0.12943	C60	1.00289	0.54965	0.62943
N7	0.42907	0.57443	0.12943	N61	0.9872	-0.05406	0.62944
C8	0.38048	0.49024	0.12943	C62	1.06678	-0.02554	0.62944
N9	0.4168	0.4423	0.12944	C63	1.10105	-0.08155	0.62944
C10	0.50099	0.47791	0.12944	C64	1.04903	-0.16817	0.62944
C11	0.54	0.42557	0.12944	C65	1.08302	-0.22047	0.62943
C12	0.28914	0.45123	0.12943	C66	1.16964	-0.18587	0.62943
C13	0.56567	0.70209	0.12943	C67	1.22194	-0.09958	0.62944

C14	0.49068	0.3386	0.12944	C68	1.18734	-0.04756	0.62944
C15	0.52762	0.28892	0.12944	N69	1.05553	0.63818	0.62943
C16	0.61423	0.32541	0.12944	C70	1.02702	-0.31074	0.62943
C17	0.66382	0.41252	0.12944	C71	1.31222	-0.06531	0.62944
C18	0.62685	0.46215	0.12943	N72	0.36329	0.01428	0.62943
C19	0.25149	0.50055	0.12942	H73	0.54826	0.87921	0.12943
C20	0.16486	0.46361	0.12942	H74	0.4584	0.7222	0.12943
C21	0.11475	0.377	0.12943	H75	0.68325	0.69964	0.12943
C22	0.15227	0.32742	0.12944	H76	0.773	0.85651	0.12943
C23	0.23887	0.36438	0.12944	H77	0.41996	0.30798	0.12945
C24	0.66582	0.87648	0.12943	H78	0.48708	0.21824	0.12945
N25	0.65013	0.27277	0.12944	H79	0.73452	0.44297	0.12943
C26	0.72971	0.30129	0.12944	H80	0.66737	0.53283	0.12943
C27	0.76398	0.24528	0.12944	H81	0.29159	0.57127	0.12942
C28	0.71196	0.15866	0.12944	H82	0.13473	0.50415	0.12941
C29	0.74595	0.10636	0.12943	H83	0.11202	0.25671	0.12945
C30	0.83257	0.14096	0.12943	H84	0.26904	0.32386	0.12945
C31	0.88487	0.22725	0.12944	H85	0.77463	0.37129	0.12944
C32	0.85027	0.27927	0.12944	H86	0.64155	0.13093	0.12944
N33	0.71846	0.96501	0.12943	H87	0.86071	0.09864	0.12943
C34	0.68995	0.01608	0.12943	H88	0.89259	0.34973	0.12944
C35	0.97515	0.26152	0.12944	H89	0.61969	-0.01059	0.12943
N36	0.02622	0.34111	0.12943	H90	1.00182	0.21794	0.12944
C37	0.91578	0.51213	0.62943	H91	0.88533	0.55238	0.62943
C38	0.86615	0.42553	0.62943	H92	0.79547	0.39537	0.62943
C39	0.9897	0.41292	0.62943	H93	1.02032	0.37281	0.62943
C40	1.03939	0.49954	0.62943	H94	1.11007	0.52968	0.62943
N41	0.886	0.23533	0.62943	H95	0.75703	-0.01885	0.62945
C42	0.8504	0.28392	0.62943	H96	1.07159	0.11614	0.62943
N43	0.76614	0.2476	0.62943	H97	1.00444	0.206	0.62943
C44	0.71755	0.16341	0.62943	H98	0.62866	0.24444	0.62942
N45	0.75387	0.11547	0.62944	H99	0.4718	0.17732	0.62941
C46	0.83806	0.15108	0.62944	H100	0.44909	-0.07012	0.62945
C47	0.87707	0.09874	0.62944	H101	0.60611	-0.00297	0.62945
C48	0.62621	0.12441	0.62943	H102	0.1117	1.04446	0.62944
C49	0.90274	0.37526	0.62943	H103	-0.02138	0.8041	0.62944
C50	0.82775	0.01177	0.62944	H104	0.19778	0.77181	0.62943
C51	0.86469	-0.03791	0.62944	H105	0.22966	1.0229	0.62944
C52	0.9513	-0.00142	0.62944	H106	-0.04324	0.66258	0.62943
C53	1.00089	0.08569	0.62944	H107	0.33889	0.89111	0.62944
C54	0.96392	0.13532	0.62943	H108	0.82415	0.89141	0.62945

Table S2. Fractional atomic coordinates for the unit cell of COF-TP.

AA stacking of COF-TP:

COF-TP: space group symmetry: P1<br>a = b = 18.6507 Å c = 3.4908 Å<br>α = β = 90 ° γ = 120 °							
N1	0.51479	-0.38102	0.50519	O30	0.83782	-0.84168	0.50519
C2	0.48146	-0.33081	0.5052	C31	0.66288	-0.92237	0.50519
N3	0.39784	-0.36486	0.50519	O32	0.59799	-0.821	0.5052
C4	0.34764	-0.44839	0.50519	C33	0.9392	-0.67541	0.50519
N5	0.38168	-0.49797	0.50519	C34	0.55263	-0.10229	0.50519
C6	0.46522	-0.46464	0.50519	C35	0.50132	-0.18823	0.50519
C7	0.50176	-0.51874	0.50519	C36	0.6219	-0.20485	0.50518
C8	0.25699	-0.48493	0.50519	C37	0.67323	-0.11889	0.50518
C9	0.53556	-0.24017	0.50519	N38	0.69274	1.02044	0.50519
C10	0.45075	-0.60507	0.50519	N39	0.99638	1.41163	0.50518
C11	0.48538	-0.65641	0.50519	H40	0.38055	-0.63379	0.50519
C12	0.5713	-0.62212	0.50519	H41	0.44349	-0.72655	0.50519
C13	0.62257	-0.5358	0.50518	H42	0.69274	-0.50732	0.50517
C14	0.58795	-0.4845	0.50518	H43	0.62982	-0.41436	0.50518
C15	0.22167	-0.43392	0.50518	H44	0.26315	-0.36373	0.50518
C16	0.13571	-0.46855	0.50518	H45	0.10745	-0.42667	0.50518
C17	0.08407	-0.55448	0.50518	H46	0.07743	-0.67592	0.50518
C18	0.11911	-0.60575	0.50518	H47	0.23331	-0.613	0.50519
C19	0.20505	-0.57112	0.50518	H48	0.56339	-0.74405	0.50519
C20	0.63894	-0.06725	0.50519	H49	0.73786	-0.57661	0.50517
N21	0.6052	-0.67592	0.50519	H50	0.59279	-0.95022	0.50519
C22	0.69224	-0.64606	0.50518	H51	0.96705	-0.71765	0.5052
C23	0.72505	-0.69582	0.50517	H52	0.52414	-0.06061	0.5052
C24	0.67344	-0.78767	0.50519	H53	0.43118	-0.21649	0.5052
C25	0.71264	-0.83981	0.50519	H54	0.65062	-0.24632	0.50518
C26	0.80449	-0.79956	0.50519	H55	0.74337	-0.09063	0.50518
C27	0.85663	-0.70822	0.50519	H56	0.76088	1.04678	0.50519
C28	0.81639	-0.65661	0.50518	H57	0.97005	1.45343	0.50517
O29	0.8585	-0.58117	0.50519				

AB stacking of COF-TP:

COF-TP: space group symmetry: P1<br>a = b = 18.6508 Å c = 6.9822 Å<br>α = β = 90 ° γ = 120 °							
N1	0.7758	0.27206	0.19082	C58	0.12752	0.41465	0.69081
C2	0.74247	0.32227	0.19082	C59	0.5614	0.91853	0.69082
N3	0.65884	0.28822	0.19082	N60	0.6152	0.00622	0.69081
C4	0.60864	0.20468	0.19082	N61	0.52767	0.30986	0.6908

N5	0.64269	0.15511	0.19082	C62	0.61471	0.33972	0.6908
C6	0.72623	0.18844	0.19082	C63	0.64751	0.28996	0.6908
C7	0.76277	0.13434	0.19082	C64	0.5959	0.19811	0.69079
C8	0.518	0.16814	0.19082	C65	0.6351	0.14597	0.6908
C9	0.79657	0.41291	0.19082	C66	0.72696	0.18621	0.69079
C10	0.71176	0.04801	0.19081	C67	0.7791	0.27755	0.6908
C11	0.74639	-0.00333	0.19081	C68	0.73885	0.32916	0.6908
C12	0.83231	0.03097	0.19081	O69	0.78097	0.40461	0.6908
C13	0.88358	0.11728	0.19081	O70	0.76029	0.14409	0.69079
C14	0.84896	0.16859	0.19082	C71	0.58535	0.0634	0.6908
C15	0.48268	0.21915	0.19082	O72	0.52046	0.16478	0.69078
C16	0.39672	0.18452	0.19081	C73	0.86166	0.31036	0.6908
C17	0.34508	0.09859	0.19081	N74	0.91885	0.3974	0.6908
C18	0.38012	0.04732	0.19081	C75	0.47509	0.88348	0.69081
C19	0.46606	0.08195	0.19081	C76	0.42378	0.79755	0.69082
C20	0.89994	0.58583	0.19082	C77	0.54436	0.78093	0.69083
N21	0.95374	-0.32648	0.19081	C78	0.59569	0.86689	0.69082
N22	0.86621	-0.02283	0.1908	H79	0.64156	0.01929	0.19081
C23	0.95325	0.00702	0.1908	H80	0.7045	-0.07346	0.1908
C24	0.98605	-0.04274	0.1908	H81	0.95375	0.14576	0.19081
C25	0.93444	-0.13459	0.19079	H82	0.89083	0.23873	0.19082
C26	0.97364	-0.18673	0.1908	H83	0.52415	0.28935	0.19082
C27	1.0655	-0.14649	0.19079	H84	0.36846	0.22641	0.19081
C28	1.11764	-0.05515	0.1908	H85	0.33844	-0.02284	0.1908
C29	1.07739	-0.00354	0.1908	H86	0.49432	0.04008	0.19081
O30	1.11951	0.07191	0.1908	H87	1.02188	-0.30015	0.19081
O31	1.09883	-0.1886	0.19079	H88	0.8244	-0.09097	0.1908
C32	0.92389	-0.26929	0.1908	H89	0.99887	0.07647	0.19081
O33	0.859	-0.16792	0.19078	H90	0.8538	-0.29714	0.1908
C34	1.2002	-0.02234	0.1908	H91	1.22805	-0.06458	0.1908
N35	1.25739	0.0647	0.1908	H92	1.23105	0.10651	0.19081
C36	0.81363	0.55079	0.19081	H93	0.78514	0.59247	0.19081
C37	0.76232	0.46485	0.19082	H94	0.69218	0.43659	0.19082
C38	0.8829	0.44823	0.19083	H95	0.91162	0.40676	0.19083
C39	0.93423	0.53419	0.19082	H96	1.00437	0.56245	0.19083
N40	0.43726	0.60476	0.69082	H97	0.30302	0.35198	0.69081
C41	0.40393	0.65496	0.69082	H98	0.36596	0.25923	0.6908
N42	0.3203	0.62091	0.69082	H99	0.61521	0.47846	0.69081
C43	0.2701	0.53738	0.69082	H100	0.55229	0.57142	0.69082
N44	0.30415	0.4878	0.69082	H101	0.18561	0.62204	0.69082
C45	0.38769	0.52114	0.69082	H102	0.02992	0.5591	0.69081
C46	0.42423	0.46704	0.69082	H103	-0.0001	0.30985	0.6908
C47	0.17946	0.50084	0.69082	H104	0.15578	0.37277	0.69081

C48	0.45803	0.7456	0.69082	H105	0.68334	0.03255	0.69081
C49	0.37322	0.3807	0.69081	H106	0.48586	0.24172	0.6908
C50	0.40785	0.32937	0.69081	H107	0.66033	0.40917	0.69081
C51	0.49377	0.36366	0.69081	H108	0.51526	0.03555	0.6908
C52	0.54504	0.44997	0.69081	H109	0.88951	0.26812	0.6908
C53	0.51042	0.50128	0.69082	H110	0.89251	0.4392	0.69081
C54	0.14414	0.55185	0.69082	H111	0.4466	0.92516	0.69081
C55	0.05818	0.51722	0.69081	H112	0.35364	0.76928	0.69082
C56	0.00654	0.43129	0.69081	H113	0.57308	0.73945	0.69083
C57	0.04158	0.38002	0.69081	H114	0.66583	0.89514	0.69083

Table S3. Fractional atomic coordinates for the unit cell of COF-TC.
AA stacking of COF-TC:

COF-TC: space group symmetry: P1<br>a = b = 18.6524 Å c = 3.4963 Å<br>α = β = 90 ° γ = 120 °							
N1	1.46217	-0.61256	-0.43589	C33	1.66437	-0.94481	-0.4359
C2	1.5458	-0.57833	-0.4359	C34	1.94218	-0.68235	-0.43589
N3	1.59458	-0.49462	-0.4359	C35	1.92684	-0.96016	-0.4359
C4	1.56035	-0.44521	-0.4359	N36	0.76892	0.90667	0.56411
N5	1.47664	-0.48014	-0.4359	C37	0.74171	0.95838	0.56411
C6	1.42723	-0.56378	-0.43589	N38	1.07536	1.29291	0.56409
C7	1.33649	-0.60128	-0.43589	C39	1.02365	1.214	0.5641
C8	1.58331	-0.63157	-0.4359	H40	1.72947	-0.35904	-0.43591
C9	1.61359	-0.35446	-0.4359	H41	1.82076	-0.2032	-0.4359
C10	1.70001	-0.31823	-0.4359	H42	1.59962	-0.17636	-0.43589
C11	1.75054	-0.23218	-0.4359	H43	1.50821	-0.33236	-0.4359
C12	1.71546	-0.18123	-0.43589	H44	1.46286	-0.74745	-0.43589
C13	1.62891	-0.21732	-0.43589	H45	1.52741	-0.83874	-0.4359
C14	1.57843	-0.30335	-0.4359	H46	1.77538	-0.6176	-0.4359
C15	1.53313	-0.71798	-0.4359	H47	1.7108	-0.52618	-0.4359
C16	1.56865	-0.76852	-0.4359	H48	1.34106	-0.48083	-0.43589
C17	1.65468	-0.73343	-0.4359	H49	1.18522	-0.54539	-0.4359
C18	1.70513	-0.64689	-0.4359	H50	1.15839	-0.79336	-0.4359
C19	1.66959	-0.59641	-0.4359	H51	1.31438	-0.72878	-0.4359
C20	1.30026	-0.5511	-0.43589	H52	1.81354	-0.69022	-0.4359
C21	1.2142	-0.58662	-0.4359	H53	1.63827	-1.01265	-0.35622
C22	1.16326	-0.67265	-0.4359	H54	1.64156	-0.94051	-0.73287
C23	1.19934	-0.72311	-0.4359	H55	1.64277	-0.91406	-0.21862
C24	1.28537	-0.68757	-0.4359	H56	1.95659	-0.65893	-0.74314
N25	1.68911	-0.7869	-0.4359	H57	2.00251	-0.66113	-0.27359
C26	1.76803	-0.75969	-0.4359	H58	1.90517	-0.65647	-0.29095
C27	1.80528	-0.81404	-0.4359	H59	1.93254	-0.97772	-0.74211

C28	1.75729	-0.90161	-0.4359	H60	1.88955	-1.01875	-0.25921
C29	1.79607	-0.95002	-0.43589	H61	1.9912	-0.92174	-0.3064
C30	1.88364	-0.91043	-0.4359	H62	0.67225	0.93443	0.56411
C31	1.93204	-0.82326	-0.4359	H63	1.0476	1.16848	0.5641
C32	1.89246	-0.77527	-0.4359				

AB stacking of COF-TC:

COF-TB: space group symmetry: P1<br>a = b = 18.6524 Å c = 6.9926 Å<br>α = β = 90 ° γ = 120 °							
N1	0.46217	0.38744	0.28205	N64	1.02319	-0.11869	0.78205
C2	0.5458	0.42167	0.28205	C65	1.10211	-0.09148	0.78205
N3	0.59458	0.50538	0.28205	C66	1.13936	-0.14584	0.78205
C4	0.56035	0.55479	0.28205	C67	1.09137	-0.23341	0.78205
N5	0.47664	0.51986	0.28205	C68	1.13015	-0.28181	0.78205
C6	0.42723	0.43622	0.28205	C69	1.21772	-0.24223	0.78205
C7	0.33649	0.39872	0.28205	C70	1.26612	-0.15505	0.78205
C8	0.58331	0.36843	0.28205	C71	1.22654	-0.10706	0.78205
C9	0.61359	0.64554	0.28205	C72	0.99845	-0.27661	0.78205
C10	0.70001	0.68177	0.28205	C73	1.27626	-0.01414	0.78205
C11	0.75054	0.76782	0.28205	C74	1.26092	-0.29195	0.78205
C12	0.71546	0.81877	0.28205	N75	1.103	0.57487	0.78205
C13	0.62891	0.78268	0.28205	C76	1.07579	0.62658	0.78205
C14	0.57843	0.69665	0.28205	N77	0.40944	-0.03888	0.78205
C15	0.53313	0.28202	0.28205	C78	0.35773	-0.1178	0.78205
C16	0.56865	0.23148	0.28205	H79	0.72947	0.64096	0.28205
C17	0.65468	0.26657	0.28205	H80	0.82076	0.7968	0.28205
C18	0.70513	0.35311	0.28205	H81	0.59962	0.82364	0.28205
C19	0.66959	0.40359	0.28205	H82	0.50821	0.66764	0.28205
C20	0.30026	0.4489	0.28205	H83	0.46286	0.25255	0.28205
C21	0.2142	0.41338	0.28205	H84	0.52741	0.16126	0.28205
C22	0.16326	0.32735	0.28205	H85	0.77538	0.3824	0.28205
C23	0.19934	0.27689	0.28205	H86	0.7108	0.47382	0.28205
C24	0.28537	0.31243	0.28205	H87	0.34106	0.51917	0.28205
N25	0.68911	0.2131	0.28205	H88	0.18522	0.45461	0.28205
C26	0.76803	0.24031	0.28205	H89	0.15839	0.20664	0.28205
C27	0.80528	0.18596	0.28205	H90	0.31438	0.27122	0.28205
C28	0.75729	0.09839	0.28205	H91	0.81354	0.30978	0.28205
C29	0.79607	0.04998	0.28205	H92	0.63827	-0.01265	0.32189
C30	0.88364	0.08957	0.28205	H93	0.64156	0.05949	0.13356
C31	0.93204	0.17674	0.28205	H94	0.64277	0.08594	0.39069
C32	0.89246	0.22473	0.28205	H95	0.95659	0.34107	0.12843
C33	0.66437	0.05519	0.28205	H96	1.00251	0.33887	0.36321
C34	0.94218	0.31765	0.28205	H97	0.90517	0.34353	0.35453

C35	0.92684	0.03984	0.28205	H98	0.93254	0.02228	0.12895
N36	0.76892	0.90667	0.28205	H99	0.88955	-0.01875	0.3704
C37	0.74171	0.95838	0.28205	H100	0.9912	0.07826	0.3468
N38	0.07536	0.29291	0.28205	H101	0.67225	0.93443	0.28205
C39	0.02365	0.214	0.28205	H102	0.0476	0.16848	0.28205
N40	0.79625	0.05565	0.78205	H103	1.06355	0.30917	0.78205
C41	0.87988	0.08987	0.78205	H104	1.15484	0.46501	0.78205
N42	0.92866	0.17359	0.78205	H105	0.9337	0.49184	0.78205
C43	0.89444	0.223	0.78205	H106	0.84229	0.33585	0.78205
N44	0.81072	0.18806	0.78205	H107	0.79694	-0.07924	0.78205
C45	0.76131	0.10443	0.78205	H108	0.86149	-0.17053	0.78205
C46	0.67057	0.06692	0.78205	H109	1.10946	0.05061	0.78205
C47	0.91739	0.03663	0.78205	H110	1.04488	0.14202	0.78205
C48	0.94768	0.31374	0.78205	H111	0.67514	0.18737	0.78205
C49	1.03409	0.34997	0.78205	H112	0.5193	0.12282	0.78205
C50	1.08462	0.43603	0.78205	H113	0.49247	-0.12516	0.78205
C51	1.04954	0.48697	0.78205	H114	0.64846	-0.06057	0.78205
C52	0.96299	0.45088	0.78205	H115	1.14762	-0.02202	0.78205
C53	0.91251	0.36486	0.78205	H116	0.97235	-0.34444	0.82189
C54	0.86721	-0.04978	0.78205	H117	0.97565	-0.27231	0.63356
C55	0.90273	-0.10031	0.78205	H118	0.97685	-0.24585	0.89069
C56	0.98876	-0.06523	0.78205	H119	1.29067	0.00928	0.62843
C57	1.03921	0.02132	0.78205	H120	1.33659	0.00707	0.86321
C58	1.00367	0.0718	0.78205	H121	1.23925	0.01174	0.85453
C59	0.63434	0.1171	0.78205	H122	1.26662	-0.30951	0.62895
C60	0.54828	0.08158	0.78205	H123	1.22363	-0.35054	0.8704
C61	0.49734	-0.00445	0.78205	H124	1.32528	-0.25353	0.8468
C62	0.53342	-0.0549	0.78205	H125	1.00633	0.60263	0.78205
C63	0.61945	-0.01936	0.78205	H126	0.38168	-0.16331	0.78205

Table S4. Drug loading of different drug loading systems

System	BZ Loading（%）	CUR Loading（%）
B@COF-TB	25.40	
B@COF-TP	28.15	
B@COF-TC	18.56	
C@COF-TB		35.17
C@COF-TP		12.96
C@COF-TC		17.00
BC@COF-TB	9.04	10.78
BC@COF-TP	9.66	3.88

BC@COF-TC	3.85	6.17
